# Malignancy-Aware Dual-Task Heatmap Decoupling for thyroid ultrasound nodule detection and classification

**DOI:** 10.3389/fonc.2026.1888077

**Published:** 2026-09-03

**Authors:** Yining Chen, Xiaxia Wang

**Affiliations:** 1 School of Information Engineering, Gansu Minzu Normal University, Hezuo, China; 2 School of Basic Medicine, Gansu University of Chinese Medicine, Lanzhou, China

**Keywords:** anchor-free object detection, feature decoupling, feature orthogonality, receptive field, thyroid nodule detection

## Abstract

**Background:**

Accurate thyroid nodule detection and benign-malignant classification on ultrasound are critical for early cancer diagnosis. Existing deep-learning detectors encode localization and classification in a shared feature space, causing inter-task feature redundancy and limiting task-specific performance.

**Methods:**

We propose Malignancy-Aware Dual-Task Heatmap Decoupling (MADTHD), a CenterNet-based framework that decouples localization and classification through asymmetric dual heads. A Position Head with small receptive fields captures precise nodule contours, while a Malignancy Head with multi-scale dilated convolutions extracts textural and boundary features indicative of benign-malignant status. A feature orthogonality loss is introduced to enforce complementary channel activation between the two heads, reducing inter-task feature redundancy. Systematic ablation studies were conducted on TN5000, and generalization was assessed via zero-shot transfer to the independent TN3K dataset.

**Results:**

MADTHD achieves a mean average precision of 0.7840 on TN5000, outperforming the CenterNet baseline by 2.61 percentage points, with the improvement most pronounced for malignant nodules. Ablation experiments demonstrate that the asymmetric dual-head architecture and the feature orthogonality loss are mutually synergistic. On the independent TN3K dataset, MADTHD maintains its relative standing against the baseline without fine-tuning.

**Conclusion:**

MADTHD provides an interpretable dual-task framework mirroring the clinical localize-then-characterize workflow. The proposed modules are lightweight and backbone-agnostic. Clinical utility remains to be established through prospective validation.

## 1 Introduction

Thyroid cancer is among the most rapidly growing malignancies worldwide in terms of incidence. According to the latest global cancer statistics released by the International Agency for Research on Cancer, the number of newly diagnosed thyroid cancer cases surged to approximately 821,000 in 2022, ranking seventh among all cancers by incidence, with a particularly pronounced burden in female populations (1). Over the past three decades, the widespread adoption of high-resolution ultrasound equipment and the implementation of large-scale population screening programs have substantially increased the detection rate of thyroid nodules; it is estimated that ultrasound examination reveals thyroid nodules in up to 60% of the general adult population (2). Given the enormous scale of this patient group, accurately differentiating malignant from benign nodules and identifying cases that genuinely warrant further intervention represent critical clinical imperatives for reducing unnecessary fine-needle aspiration biopsies, minimizing healthcare resource consumption, and improving patient outcomes. Owing to its absence of ionizing radiation, operational convenience, and real-time dynamic imaging capability, ultrasound has become the modality of choice for initial thyroid nodule screening and diagnosis, and is extensively utilized in clinical practice. The Thyroid Imaging Reporting and Data System (TI-RADS), published by the American College of Radiology, is currently the most widely adopted international standard for ultrasound-based risk stratification of thyroid nodules, with its core assessment dimensions encompassing nodule composition, echogenicity, shape, margin, and echogenic foci (3). Nevertheless, inherent characteristics of ultrasound imaging, including strong noise, severe speckle artifacts, and indistinct nodule boundaries, present substantial diagnostic challenges (4).

Consequently, even experienced radiologists and sonographers face considerable subjective diagnostic uncertainty in certain cases. Studies have demonstrated significant interobserver variability in TI-RADS grading of the same nodule, with both overdiagnosis and overtreatment remaining non-negligible (5). Therefore, developing artificial intelligence (AI)-based systems for automated thyroid ultrasound analysis, capable of providing objective, consistent, and high-precision nodule detection and malignancy differentiation, has emerged as an important research topic in the field of medical imaging AI (6).

Deep learning-driven object detection has achieved remarkable progress in automated medical image analysis. Two-stage anchor-based approaches, exemplified by Faster R-CNN (7), leverage region proposal networks to extract regions of interest with high precision, yet their inference speed is inherently constrained. Single-stage approaches, such as the YOLO series (8), achieve an effective balance between speed and accuracy through end-to-end regression, and have been widely adopted for medical object detection tasks. In recent years, anchor-free detection paradigms have gained increasing prominence. Among these, CenterNet (9) reformulates object detection as a keypoint heatmap estimation problem targeting object center points, achieving efficient and accurate object localization through the simultaneous prediction of center point heatmaps, object dimensions, and center offsets. This streamlined pipeline eliminates the need for predefined multi-scale anchor boxes and obviates the complexity of hyperparameter tuning, conferring particular advantages in the detection of morphologically irregular medical lesions (10). However, directly applying CenterNet to the joint detection and malignancy classification of thyroid nodules reveals a critical structural limitation: in the original CenterNet framework, spatial localization information and categorical semantic information share a common convolutional feature extraction pathway, with both modalities being mixed and encoded within a unified heatmap feature space. For thyroid nodule detection and malignancy classification, the localization task, accurately identifying nodule center points, primarily relies on the geometric characteristics of nodule contours and the spatial gradients of echogenic boundaries, whereas the malignancy differentiation task depends more heavily on fine-grained semantic features such as internal textural heterogeneity, the spatial distribution of microcalcification foci, and the degree of margin spiculation (11). These two categories of features differ fundamentally in their spatial frequency properties, required receptive field (RF) scales, and gradient optimization directions. Forcing them to share a unified feature extraction pathway induces mutual interference between gradient signals and promotes negative transfer between tasks, thereby constraining the model’s capacity for joint optimization and limiting the overall ceiling of detection performance. Furthermore, the majority of existing deep learning methods treat detection and classification as a tightly coupled problem, lacking explicit modeling of the inherent heterogeneity between these two task types. Such coupled designs struggle to accommodate the distinct characteristics of each task when confronted with complex data distributions involving small-volume nodules or morphologically irregular nodules, resulting in substantial trade-offs between recall and precision.

To address these limitations, this work proposes the Malignancy-Aware Dual-Task Heatmap Decoupling (MADTHD) framework. Building upon CenterNet as the baseline architecture, MADTHD introduces explicit feature-level decoupling between the localization and malignancy differentiation tasks. The principal contributions are summarized as follows:

1. A clinically motivated, asymmetric dual-task decoupling head. We do not claim that decoupled detection heads are themselves novel; they are well established (e.g., the decoupled head of YOLOX (12), the classification/centerness branches of FCOS (13), and Double-Head R-CNN (14)). Our contribution lies in the specific form of decoupling. First, MADTHD re-draws the decoupling boundary along the clinical “localize-then-characterize” axis rather than the conventional classification/regression axis: a class-agnostic Position Head jointly predicts a single-channel nodule-presence heatmap together with size and offset regression, while a separate Malignancy Head predicts only the benign/malignant heatmap. Second, whereas decoupled heads are usually instantiated as two branches of the same form, and designs that do differentiate the branches, such as Double-Head R-CNN (14), differentiate the operator type rather than specifically differentiating the receptive field, the two heads of MADTHD are asymmetric in receptive field: the Position Head uses small-receptive-field (3 × 3) convolutions for spatially precise localization, whereas the Malignancy Head uses multi-scale dilated convolutions to enlarge the receptive field for peripheral malignancy cues. This asymmetry is driven by quantitative dataset statistics (small-object dominance and the larger spatial extent of malignancy features) and addresses a receptive-field trade-off that a single shared configuration cannot accommodate.
2. An explicit inter-branch feature-decorrelation constraint. Structural branching alone does not prevent the two branches from learning redundant, highly correlated features; we show empirically that the inter-branch feature similarity remains close to its maximum without an explicit constraint. MADTHD therefore introduces a feature-decorrelation regularizer that acts directly on the head-specific feature maps, driving them toward orthogonality and yielding two interpretable, less redundant heatmaps (“where” vs. “what”).
3. A systematic empirical study on the TN5000 dataset (15). This study quantifies the contribution of each architectural component, the sensitivity of the decoupling strength, and the resulting feature decorrelation, providing design guidance for dual-task decoupling in medical lesion detection.

## 2 Related work

### 2.1 Thyroid ultrasound nodule detection and classification

The evolution of computer-aided analysis for thyroid ultrasound nodules can be broadly divided into two major phases: traditional image processing and deep learning. In the traditional phase, researchers primarily relied on classical image segmentation techniques, such as active contour models (16) and level set methods, to delineate nodule boundaries, followed by extraction of hand-crafted shape, texture, and frequency-domain features for benign-malignant classification (17). These approaches yielded acceptable performance under idealized conditions with high image quality and well-defined nodule boundaries; however, they exhibited considerable sensitivity to the inherent speckle noise and boundary ambiguity of ultrasound images, resulting in insufficient robustness for large-scale deployment in real clinical settings.

The advent of deep learning has driven rapid advances in thyroid ultrasound analysis. In the domain of segmentation, Ma et al. (18) developed and evaluated a cascaded deep convolutional neural network for fully automated thyroid nodule detection from two-dimensional ultrasound images. Yang et al. (19) proposed a model based on the Swin U-Net architecture combined with self-attention mechanisms, which improved the accuracy of thyroid nodule segmentation. Zhang et al. (20) cascaded UNet and CH-UNet to simultaneously segment thyroid nodules and distinguish between benign and malignant cases. In the domain of classification, Xu et al. (21) investigated the predictive performance of both conventional machine learning algorithms and deep transfer learning models for differentiating benign from malignant thyroid nodules. Han et al. (22) incorporated clinical prior knowledge by introducing TI-RADS scoring criteria as auxiliary supervision signals into a deep classification network, achieving an organic integration of knowledge-driven and data-driven approaches, and proposed an efficient, interpretable end-to-end thyroid nodule classification network. Zhang et al. (23) further improved the recognition accuracy of malignant nodules with irregular margins by modeling the topological structure of thyroid nodules via graph convolutional networks. In the area of object detection, Zhang et al. released the TN5000 dataset (15), comprising 5,000 ultrasound images sourced from the Cancer Hospital of the Chinese Academy of Medical Sciences, with fine-grained bounding box annotations for both benign and malignant nodules, thereby providing a data foundation for large-scale joint detection and differentiation research.

### 2.2 Keypoint-based object detection

Anchor-free object detection has emerged as an important research direction in recent years, aiming to completely eliminate the strong prior dependence of conventional anchor mechanisms on predefined scales and aspect ratios. CornerNet (24) pioneered the reformulation of object detection as a corner-pair detection problem, predicting heatmaps for the top-left and bottom-right key points of each object and subsequently matching corner pairs belonging to the same instance via an associative embedding module, thereby achieving fully anchor-free detection. Building upon this foundation, CenterNet (9) further simplified the detection problem to single-keypoint estimation at the object center, representing each object solely by its center coordinates, width, height, and center offset. This design substantially reduced model complexity and improved inference speed, establishing CenterNet as a prominent baseline in the anchor-free detection literature. FCOS (13) adopted a per-pixel prediction paradigm, directly regressing the distances from each pixel to the four sides of the target bounding box and incorporating a centerness prediction branch to suppress low-quality candidate boxes, thereby achieving high-accuracy anchor-free detection without reliance on keypoint heatmaps.

Subsequent work extended these anchor-free frameworks in multiple directions. CenterNet2 (25) introduced a cascaded two-stage design in which first-stage center point hypotheses guided second-stage refined detection, substantially improving accuracy while preserving the advantages of the anchor-free paradigm. GFL (26) proposed generalized focal loss, extending discrete label supervision to continuous distribution modeling and improving the consistency between bounding box quality estimation and heatmap response. ATSS (27) eliminated the inconsistency between classification and regression tasks through an adaptive training sample selection strategy that redefines positive sample assignment. Furthermore, DETR (28) introduced the transformer architecture into object detection, modeling global contextual relationships via self-attention mechanisms and offering a new research direction for the advancement of detection paradigms.

In the medical imaging domain, CenterNet-based detection frameworks have been applied to the automatic detection of various lesion types. Gong et al. (29) drew inspiration from keypoint estimation methods and proposed an anchor-free framework for pulmonary nodule detection, validating the applicability of anchor-free approaches in medical image scenarios. Luo et al. (30) further addressed the challenge of large-scale variation in medical lesions by proposing SCPM-Net, an anchor-free detection network based on center point matching, which significantly improved the spatial localization accuracy of irregularly shaped nodules. Collectively, these studies demonstrate that CenterNet possesses strong cross-modal transferability and serves as a powerful foundational framework for medical object detection research. Nevertheless, most existing detection methods do not explicitly distinguish between the heterogeneous tasks of localization and differentiation, so the features serving the two tasks remain entangled, which is the problem the present work addresses.

### 2.3 Multi-task learning and feature decoupling

Multi-task learning (31) simultaneously optimizes multiple related tasks by exploiting shared information across them, offering superior sample efficiency and generalization capability compared to independent single-task training. It has been widely adopted in object detection, scene understanding, and medical image analysis. Within object detection frameworks, classification and regression are naturally treated as two sub-tasks. Kendall et al. (32) proposed a multi-task loss balancing method based on homoscedastic uncertainty, employing learnable task-weight parameters to automatically adjust the relative contribution of each task loss, effectively alleviating the burden of manual weight tuning and demonstrating performance gains on multiple detection benchmarks. GradNorm (33) addressed gradient imbalance in multi-task training from the perspective of gradient magnitude equalization, dynamically adjusting per-task gradient normalization coefficients to promote balanced optimization. However, one of the core challenges in multi-task learning is negative transfer: when the gradient directions of different tasks conflict, the learning signal from one task may interfere with or even suppress the optimization of another, causing joint training to underperform independent single-task training (34). To mitigate this issue, feature decoupling strategies have been proposed, with the central idea of suppressing task conflicts by explicitly separating the feature representations dedicated to different tasks. PAA (35) reexamined the sample assignment problem from a probabilistic perspective, unifying the optimization objectives of classification and localization through a probabilistic positive sample assignment mechanism to further improve task synergy. OTA (36) applied optimal transport theory to globally optimize positive sample assignment, effectively alleviating the conflict between classification confidence and localization quality from an information-matching perspective.

In the field of representation learning, mutual information minimization is an important theoretical tool for achieving statistical independence between feature representations. The mutual information neural estimation method proposed by Belghazi et al. (37) estimates a differentiable lower bound of mutual information via a trainable neural network, enabling the mutual information minimization objective to be incorporated end-to-end into deep network training. Chen et al. (38) interpreted the representation learning objective in the contrastive self-supervised framework SimCLR from the perspective of mutual information maximization, elucidating the critical roles of data augmentation strategies and projection heads in enhancing feature quality, and establishing a theoretical basis for feature decoupling. In medical image analysis, Chartsias et al. (39) applied the feature decoupling paradigm to cardiac MRI analysis and proposed the spatial decomposition network, which disentangles two-dimensional medical images into spatial anatomical factors and non-spatial modality factors.

The value of inter-task feature decoupling in multi-task medical image detection has become increasingly prominent. In YOLOX, Ge et al. (12) conducted an in-depth analysis of the spatial response conflicts between classification and regression tasks, and introduced a physically isolated decoupled head into a single-stage anchor-free detector, demonstrating that the separation of feature extraction pathways substantially accelerates model convergence and improves detection accuracy, providing important structural design guidance for multi-task networks. Furthermore, Cheng et al. (40) proposed the CLUB estimator, applying mutual information minimization as an effective regularization technique in deep neural networks to successfully suppress redundant correlations between features of different tasks, providing a rigorous mathematical optimization tool for feature orthogonalization. Collectively, these works demonstrate that explicitly modeling task heterogeneity and implementing targeted feature decoupling strategies constitutes an effective approach to improving the performance of multi-task medical image analysis.

A parallel line of work in medical imaging combines convolutional and attention-based architectures and benchmarks them systematically. Such studies fuse convolutional and transformer backbones for lesion-level binary classification (41), compare pre-trained CNNs with vision transformers on a common dermatological benchmark (42), and compare learning-based predictors on structured clinical data (43). These studies address backbone selection and classification benchmarking, and are therefore complementary to the head-level question examined here.

In contrast to these works, MADTHD differs from existing decoupled detectors along three methodological dimensions rather than by mere domain transfer. (i) Decoupling axis: conventional decoupled heads separate classification from bounding-box regression, whereas MADTHD separates class-agnostic localization (presence heatmap, size, and offset) from benign/malignant discrimination, mirroring the clinical detect-then-diagnose workflow and introducing an explicit class-agnostic presence heatmap. (ii) Branch design: MADTHD deliberately employs asymmetric branches whose receptive fields are matched to each task and justified by the scale statistics of the dataset. (iii) Feature regularization: structural separation is the common form of decoupling, and additional constraints, where they are used, act on proposals or predictions (44); MADTHD instead penalizes the similarity of the two head-level feature descriptors directly.

## 3 Materials and methods

### 3.1 Dataset

#### 3.1.1 Dataset overview

This study employed the TN5000 thyroid nodule ultrasound dataset (15) as the experimental benchmark. The dataset comprises 5,000 clinical thyroid ultrasound images sourced from the Cancer Hospital of the Chinese Academy of Medical Sciences. All images were annotated by board-certified radiologists, and each image contains exactly one nodule target. Annotations follow the VOC XML bounding-box format, with two classes: benign nodule (BN, class 0) and malignant nodule (MN, class 1), yielding a total of 5,000 annotated bounding boxes. The official data split protocol was adopted, partitioning the dataset into a training set (3,500 images), a validation set (500 images), and a test set (1,000 images) in a 7:1:2 ratio. We adopted the exact official split released together with the publicly downloadable TN5000 benchmark dataset: the source data are already assigned to the training, validation, and test subsets, and these pre-assigned subsets were used directly, without any modification on our part. We adopted this official partition rather than constructing a custom split, so that our results remain directly comparable with other methods evaluated on the same benchmark and are not affected by partition-selection bias. This choice is further consistent with common practice in the field: the 70%/10%/20% train/validation/test proportion is a widely used convention in object detection and medical image analysis, providing a sufficiently large training set while reserving an independent validation set for model selection and a held-out test set for unbiased evaluation.

Image resolution is predominantly 718 × 500 pixels, with widths ranging from 439 to 919 pixels and heights ranging from 349 to 646 pixels. Pixel-level statistics computed over a 500-image sample revealed a mean grayscale intensity of 60.04 ± 17.74 and a pixel standard deviation (std) of 45.68 ± 7.86. The mean speckle noise index is 0.829 ± 0.263, substantially higher than that of natural image datasets, reflecting the inherent severe speckle interference characteristics of ultrasound imaging. **Table 1** consolidates the principal descriptive statistics of the dataset.

**Table 1:** Descriptive statistics of the TN5000 dataset.

Characteristic	Value
Total images/annotated nodules	5,000/5,000
Classes	Benign (BN, class 0), Malignant (MN, class 1)
Benign: Malignant ratio	≈ 1: 2.5
Official split (train/val/test)	3,500/500/1,000 (7:1:2)
Native image resolution	predominantly 718 × 500 px (width 439–919, height 349–646)
Mean grayscale intensity	60.04 ± 17.74
Mean pixel standard deviation	45.68 ± 7.86
Mean speckle-noise index	0.829 ± 0.263
Relative nodule area (mean ± std)	6.4% ± 8.6%
Size distribution (small/medium/large)	4,150 (83.0%)/825 (16.5%)/25 (0.5%)
Nodule aspect ratio (mean ± std; range)	1.23 ± 0.35; 0.43–3.34

#### 3.1.2 Analysis of key dataset characteristics

Through quantitative analysis, the two dimensions of class imbalance and object scale distribution were examined to reveal the key characteristics of the TN5000 dataset, from which the data-driven motivation for the dual-head decoupled design of MADTHD is derived.

Severe class imbalance: A dedicated classification head is required for benign-malignant discrimination.

The benign-to-malignant sample ratio in TN5000 is approximately 1:2.5, indicating pronounced class imbalance. This imbalance imposes two direct design constraints. First, the learning of classification heatmaps is subject to a prior-bias problem. In the original CenterNet, localization and classification share a common feature pathway. Under severely imbalanced class conditions, the gradient signal from the dominant malignant class exerts a disproportionately stronger shaping influence on the shared features during back-propagation, biasing the shared representation toward malignant nodule recognition and suppressing the learning of localization features for benign nodules, which consequently reduces the recall rate for benign nodules. Decoupling position localization from class classification into independent feature pathways allows the Position Head to learn general nodule contour features from a class-agnostic perspective, thereby reducing the interference of class imbalance on localization feature learning. Second, accurate discrimination of malignant nodules requires a dedicated classification feature learning pathway. The primary diagnostic features of malignant nodules, typically manifesting as punctate calcifications, spiculated margins, and taller-than-wide morphology, are visually distinct in nature from those of benign nodules, which tend to present as rounded, well-defined, and homogeneously echogenic (45, 46). To enable the network to fully learn these class-discriminative features without interference from localization task gradients, an independent Malignancy Head is designed to focus exclusively on the extraction of class-sensitive features. Furthermore, to address training instability induced by class imbalance, both task heads employ focal loss as the supervisory objective, dynamically down-weighting high-confidence easy samples to amplify the learning weight of minority-class instances and mitigate the adverse effects of class imbalance.

Small-object dominance: Differential receptive fields required for localization and discrimination.

**Figure 1:**
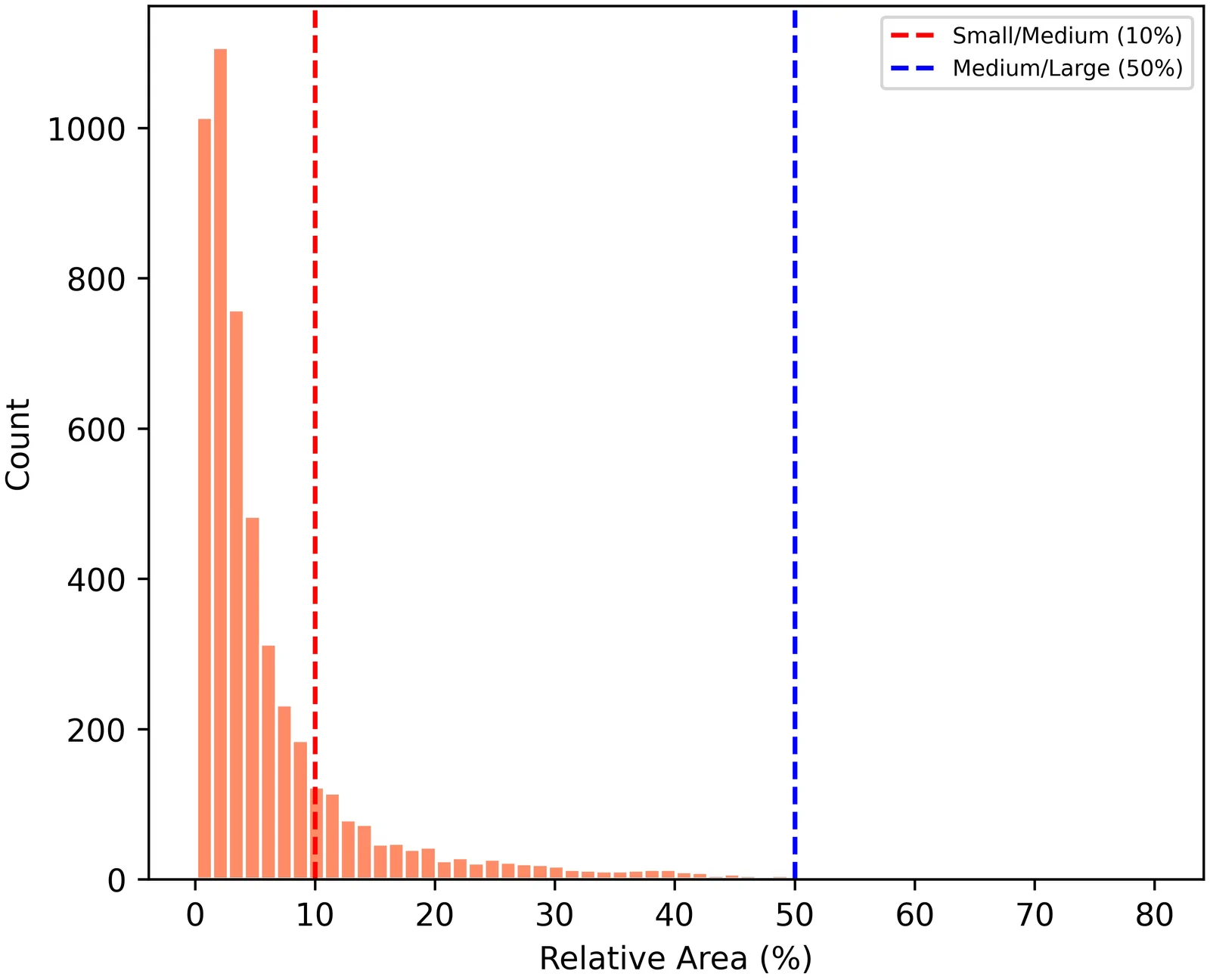
Histogram of relative nodule area distribution. The vertical axis represents sample count, and the horizontal axis represents nodule area as a percentage of total image area. Two vertical dashed lines denote the thresholds separating small from medium objects (10%) and medium from large objects (50%), respectively.

**Figure 1** presents the histogram of relative nodule bounding-box area in TN5000. The statistical results show that 83.0% of nodule instances are small objects (relative area <10%, 4,150 cases), 16.5% are medium objects (relative area 10%–50%, 825 cases), and only 0.5% are large objects (relative area ≥50%, 25 cases). The mean relative nodule area is 6.4% (standard deviation 8.6%). This highly concentrated small-object distribution imposes differentiated requirements on the receptive field design of the detection head and constitutes the core data-driven motivation for the dual-head receptive field decoupling design in MADTHD.

For the localization task (Position Head): precise localization of small objects demands fine spatial resolution awareness. An excessively large receptive field causes spatial averaging over small-object regions, shifting the heatmap peak position and degrading center-point localization accuracy. Accordingly, the Position Head employs two cascaded 3 × 3 standard convolution layers (equivalent receptive field of 5 × 5) to preserve precise spatial boundary information at the scale of small nodules, thereby generating sharp Gaussian response peaks at small-object centers in heatmap space.

For the benign/malignant discrimination task (Malignancy Head): the key diagnostic cues of malignant nodules are typically distributed over a spatial extent considerably larger than the nodule center alone. Relying solely on a small receptive field focused on local central texture is insufficient to capture critical peripheral pathological features such as irregular margins. To address this, the Malignancy Head incorporates dilated convolutions with dilation rates *r* = 2 and *r* = 4; combined with the preceding base convolution layer, the two branches yield cumulative receptive fields of 7×7 and 11×11, respectively, expanding the receptive field without reducing feature map resolution. As a result, the discriminative features encompass the complete spatial extent from the nodule center to its periphery, enhancing the ability to capture morphological features characteristic of malignancy. The intentional differentiation of receptive fields between the Position Head and the Malignancy Head is precisely driven by the data characteristics described above: the dominance of small objects and the inconsistent spatial distribution ranges of benign versus malignant features. If both tasks shared a common receptive field design, a compromise between precise localization (which requires a small receptive field) and thorough discrimination (which requires a large receptive field) would be inevitable, preventing either task from reaching its optimal performance. MADTHD eliminates this conflict through dual-head decoupling, matching each task with the feature extraction strategy most suited to its requirements.

### 3.2 Overall framework

**Figure 2:**
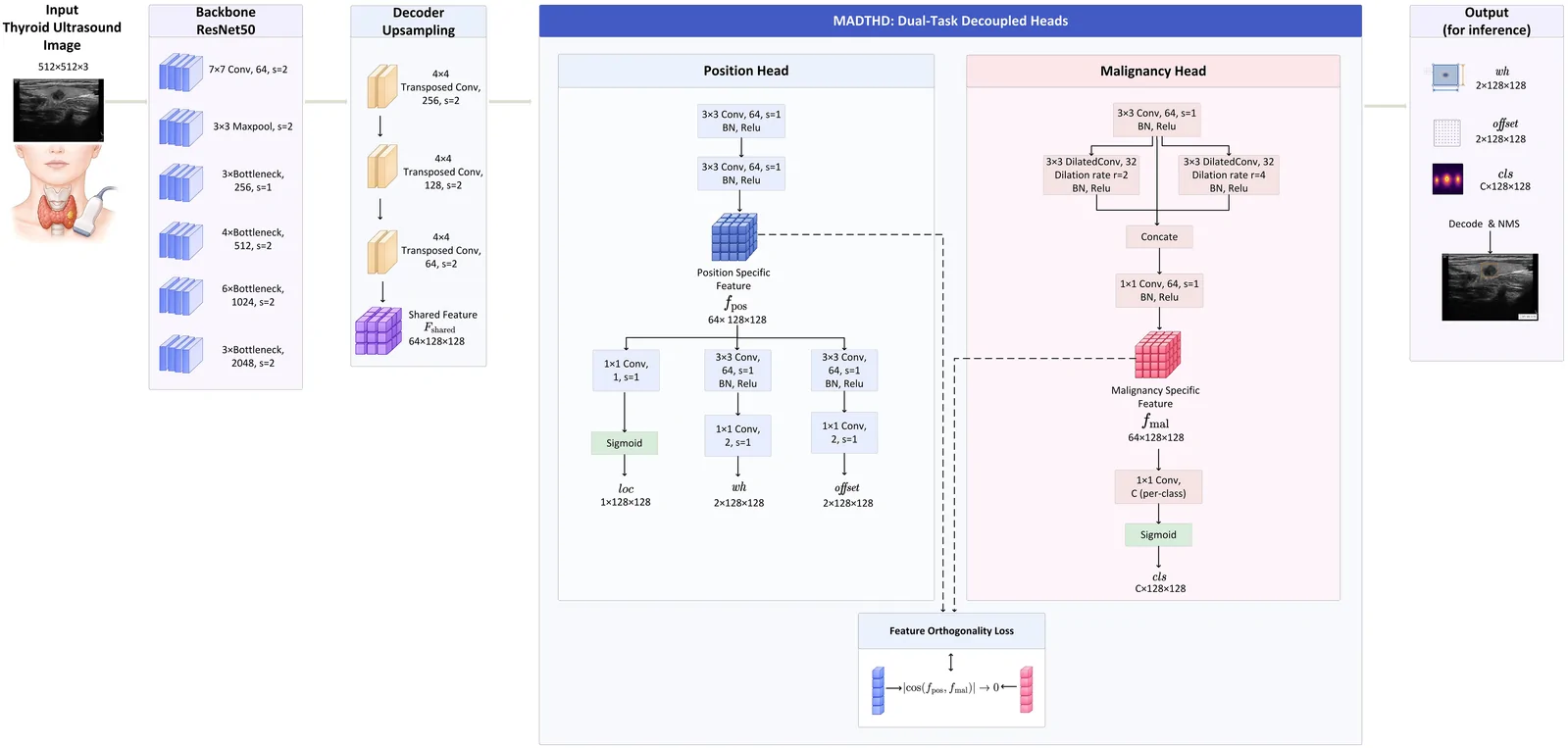
Overall architecture of MADTHD for thyroid ultrasound nodule detection and classification.

**Figure 2** presents the overall architecture of MADTHD for thyroid ultrasound nodule detection and malignancy classification. The entire network adopts CenterNet as the baseline framework, upon which the detection head is systematically restructured to incorporate a task-specific dual-head decoupled design. Shared backbone. A ResNet50 (47) pretrained on ImageNet is employed as the feature extractor. Its four-stage residual structure hierarchically extracts multi-scale visual features ranging from low-level textures to high-level semantics, ultimately producing a high-semantic feature map with spatial dimensions equal to 1/32 of the original input, denoted as *F*_backbone_ ∈ R*^B^*^×2048×16×16^, where *B* represents the batch size, given a 512 × 512 input.

Shared decoder. Composed of three cascaded transposed convolution layers (each with stride 2), this module progressively upsamples the 16 × 16 backbone output to 32 × 32, 64 × 64, and 128 × 128, yielding a 64-channel shared feature map *F*_shared_ ∈ R*^B^*^×64×128×128^, restoring spatial resolution while preserving high-level semantic information.

Dual-task decoupled heads. Consisting of a Position Head and a Malignancy Head operating in parallel, both receive the same shared feature map *F*_shared_ as input. Each head extracts task-specific feature representations through its own separate convolutional modules and outputs the corresponding prediction heatmap.

The core design rationale of this architecture lies in explicitly decomposing the two sub-problems central to thyroid nodule diagnosis, “where is the nodule” (localization) and “what type is the nodule” (benign/malignant discrimination), into separate network branches, each with fundamentally different feature dependencies. The dual-head architecture equips each task with a dedicated convolutional module matched to its feature requirements, such that spatial localization and malignancy discrimination share feature learning in the early layers while diverging in the deeper layers, realizing a strategy of limited sharing combined with deep specialization. The feature orthogonality loss acts on top of this structural separation by penalizing the similarity of the two task-specific feature descriptors, so that the two branches are encouraged to occupy complementary directions in channel space. Let **x** denote an input image batch.

The overall forward propagation process is formalized by Equations 1–4 as follows:

(1) $$F_{\text{backbone}}=\text{Backbone }(x)\in {\mathbb{R}}^{B\times 2048\times 16\times 16}$$

(2) $$F_{\text{shared}}=\text{Decoder }(F_{\text{backbone}})\in {\mathbb{R}}^{B\times 64\times 128\times 128}$$

(3) $$\left(loc,wh,offset,f_{pos})=\text{PositionHead }(F_{\text{shared}}\right)$$

(4) $$\left(cls,f_{mal})=\text{MalignancyHead }(F_{\text{shared}}\right)$$

During the training phase, both task-specific heads and the feature orthogonality loss $L_{\text{orth}}$ are simultaneously activated, and the network outputs a complete six-tuple $\left(loc,cls,wh,offset,f_{\text{pos}},f_{\text{mal}}\right)$. Among these, $f_{\text{pos}}$ and $f_{\text{mal}}$ participate exclusively in the computation of $L_{\text{orth}}$, promoting decorrelation between the two feature streams via a cosine-similarity constraint. The localization heatmap loc is trained with an auxiliary position-specific focal loss ( $L_{\text{pos}}$) but is discarded at inference. cls serves as the primary classification output, while wh and offset provide the box-size and center-offset regressions. At inference, only cls, wh and offset are used for decoding.

### 3.3 Position head

The Position Head detects the spatial locations of all nodules independently of category, using stacked small-receptive-field convolutions to capture contour and center information. As illustrated in **Figure 2**, the Position Head receives the shared feature map $F_{\text{shared}}\in {\mathbb{R}}^{B\times 64\times 128\times 128}$ output by the decoder. To extract higher-purity localization semantics, two consecutive 3×3 convolutional layers are first applied to refine the features, producing the task-specific localization feature map $f_{\text{pos}}$, as formalized in Equation 5, where $f_{\text{pos}}\in {\mathbb{R}}^{B\times 64\times 128\times 128}$, ${\text{Conv}}_{3\text{ x }3}$ denotes a 2D convolutional layer with a 3×3 kernel, BN denotes Batch Normalization, and ReLU denotes the Rectified Linear Unit activation function.

(5) $$f_{\text{pos}}=\text{ReLU }(\text{BN }({\text{Conv}}_{3\text{ x }3}(\text{ReLU }(\text{BN }({\text{Conv}}_{3\text{ x }3}(F_{\text{shared}}))))))\in {\mathbb{R}}^{B\times 64\times 128\times 128}$$

Subsequently, *f*_pos_ is fed into three parallel sub-task branches to achieve end-to-end nodule localization, as defined in Equations 6, 7.

Center-point heatmap prediction. This branch reduces the channel dimension to 1 via a 1 × 1 convolution, followed by a sigmoid activation function to produce the localization heatmap $\;loc\in {\left[0,1\right]}^{B\times 1\times 128\times 128}$.

Object scale regression. This branch regresses the predicted bounding-box width and height of each nodule through a combination of one 3 × 3 convolutional layer and one 1 × 1 convolutional layer, where *wh* $\in {\mathbb{R}}^{B\times 2\times 128\times 128}$.

(6) $$wh={\text{Conv}}_{1\text{ x }1}(\text{ReLU }(\text{BN }({\text{Conv}}_{3\text{ x }3}(f_{\text{pos}}))))$$

Center-point offset compensation. To correct the coordinate quantization error introduced by the downsampling in the backbone network, this branch regresses the center-point offset. By incorporating this offset compensation, the model can more accurately map predicted coordinates on the feature map back to the original ultrasound image.

(7) $$offset={\text{Conv}}_{1\text{ x }1}(\text{ReLU }(\text{BN }({\text{Conv}}_{3\text{ x }3}(f_{\text{pos}}))))$$

### 3.4 Malignancy head

The Malignancy Head is responsible for predicting the benign/malignant category at each nodule center pixel and adopts a multi-scale dilated convolution fusion design: standard convolutions extract baseline semantic features, while dilated convolutions with dilation rates *r* = 2 and *r* = 4, combined with the preceding base convolution, yield cumulative receptive fields of 7 × 7 and 11 × 11, respectively. The three-branch feature fusion balances local fine-grained texture with large-scale boundary morphology, as formulated in Equations 8–12, where [base;d2;d4] denotes channel-wise concatenation (64 + 32 + 32 = 128 channels), which is subsequently compressed to 64 channels via a 1 × 1 convolution to yield the malignancy discriminative feature *f*_mal_, where *C* denotes the number of target classes (in this study, *C* = 2, corresponding to benign and malignant nodules).

(8) $$base=\text{ReLU }(\text{BN }({\text{Conv}}_{3\text{ x }3}(F_{\text{shared}})))\in {\mathbb{R}}^{B\times 64\times 128\times 128}$$

(9) $$d_{2}=\text{ReLU }(\text{BN }({\text{DilatedConv}}_{3\text{ x }3,\;r=2}(\text{base})))\in {\mathbb{R}}^{B\times 32\times 128\times 128}$$

(10) $$d_{4}=\text{ReLU }(\text{BN }({\text{DilatedConv}}_{3\text{ x }3,\;r=4}(\text{base})))\in {\mathbb{R}}^{B\times 32\times 128\times 128}$$

(11) $$f_{\text{mal}}=\text{ReLU }(\text{BN }({\text{Conv}}_{1\text{ x }1}([\text{base};d_{2};d_{4}])))\in {\mathbb{R}}^{B\times 64\times 128\times 128}$$

(12) $$cls=\text{Sigmoid }({\text{Conv}}_{1\text{ x }1}(f_{\text{mal}}))\in [0,1{]}^{B\times C\times 128\times 128}$$

### 3.5 Loss function design

The MADTHD framework augments the original regression losses of CenterNet with a position-specific focal loss and a feature orthogonality loss, constructing a multi-task joint optimization objective tailored for benign/malignant classification of thyroid nodules. The total loss function is defined as Equation 13:

(13) $$L_{\text{total}}=L_{\text{pos}}+L_{\text{mal}}+\alpha \cdot L_{\text{wh}}+L_{\text{off}}+\lambda_{\text{orth}}\cdot L_{\text{orth}}$$

#### 3.5.1 Position focal loss $L_{\text{pos}}$

To constrain the Position Head to focus on learning class-agnostic nodule presence, the ground-truth Gaussian heatmaps of all classes are aggregated along the channel dimension via a pixel-wise maximum operation to construct a single-channel, class-agnostic location target heatmap. The formula is given in Equation 14, where ${\hat{Y}}_{c}(i,j)$ denotes the Gaussian ground-truth response of class c at heatmap position (i,j), and ${\hat{Y}}_{\text{pos}}(i,j)$ takes the maximum over all class responses to eliminate class-specific information.

(14) $${\hat{Y}}_{\text{pos}}(i,j)=\underset{c}{\max}{\hat{Y}}_{c}(i,j)$$

Following the focal loss formulation proposed in CornerNet, modulation weights are applied to the position prediction heatmap to suppress the large number of negative samples in background regions, while reducing the penalty for difficult samples near object centers. The formula is given in Equation 15, where ${\hat{p}}_{ij}$ is the position confidence at feature map location (*i,j*) output by the Position Head after Sigmoid activation, and *N*_pos_ is the total number of positive object center points in the current batch, used for normalization.

(15) $$L_{\text{pos}}=-\frac{1}{N_{pos}}\sum_{i,j}\{\begin{array}{ll} {\left(1-{\hat{p}}_{ij}\right)}^{2}\text{log }({\hat{p}}_{ij}), & {\hat{Y}}_{\text{pos}}(i,j)=1\\ {\left(1-{\hat{Y}}_{\text{pos}}(i,j)\right)}^{4}{\hat{p}}_{ij}^{2}\text{log }(1-{\hat{p}}_{ij}), & \text{otherwise} \end{array}$$

#### 3.5.2 Malignancy classification focal loss $L_{\text{mal}}$

The Malignancy Head is supervised using the multi-class focal loss consistent with the original CenterNet framework. Unlike $L_{\text{pos}}$, which eliminates class information, $L_{\text{mal}}$ retains independent channels for each class in the target heatmap, thereby driving this branch to learn discriminative texture and boundary features that differentiate benign from malignant nodules. The formula is given in Equation 16, where ${\hat{q}}_{cij}$ is the predicted probability of class *c* at position (*i,j*) output by the Malignancy Head, and *C* is the total number of classes.

(16) $$L_{\text{mal}}=-\frac{1}{N_{pos}}\sum_{c=1}^{C}\sum_{i,j}\{\begin{array}{ll} {\left(1-{\hat{q}}_{cij}\right)}^{2}\text{log }({\hat{q}}_{cij}), & {\hat{Y}}_{c}(i,j)=1\\ {\left(1-{\hat{Y}}_{c}(i,j)\right)}^{4}{\hat{q}}_{cij}^{2}\text{log }(1-{\hat{q}}_{cij}), & \text{otherwise} \end{array}$$

#### 3.5.3 Regression losses $L_{\text{wh}}$ and $L_{\text{off}}$

Both the width/height regression loss and the center-point offset regression loss are supervised by the corresponding branches of the Position Head, using the $L_{1}$ loss formulation. These formulas are given in Equations 17, 18 where M(i,j) is the object center mask matrix; ${\hat{s}}_{ij}$ and $s_{ij}$ denote the predicted and ground-truth bounding-box width/height, respectively; and ${\hat{\delta }}_{ij}$ and $\delta_{ij}$ denote the predicted and ground-truthcenter offset residuals, respectively. The width/height loss weight α=0.1 balances the contribution of wh to the total loss, preventing it from dominating the gradient direction. The offset loss compensates for coordinate quantization errors introduced by the heatmap downsampling stride, thereby improving the localization accuracy of the final predicted bounding boxes.

(17) $$L_{\text{wh}}=\frac{1}{N_{pos}}\sum_{i,j}M(i,j){\|\hat{s}}_{ij}-s_{ij}{\|}_{1}$$

(18) $$L_{\text{off}}=\frac{1}{N_{pos}}\sum_{i,j}M(i,j)\|{\hat{\delta }}_{ij}-\delta_{ij}{\|}_{1}$$

#### 3.5.4 Feature orthogonality loss $L_{\text{orth}}$

Although the dual-head architecture structurally bifurcates the feature pathways, the two heads still share a common input $F_{\text{shared}}$ and can learn redundant representations. To suppress this redundancy, we introduce a feature orthogonality loss that drives the two task-specific descriptors toward complementary channel activation. The formula is given in Equation 19, for each image, Global Average Pooling compresses the task-specific feature maps into channel vectors $z_{\text{pos}}=\text{GAP}(f_{\text{pos}})$ and $z_{\text{mal}}=\text{GAP}(f_{\text{mal}})$, which are $L_{2}$-normalized. The loss is the batch-mean cosine similarity:

(19) $$L_{\text{orth}}=\frac{1}{B}\sum_{i=1}^{B}|\frac{z_{\text{pos},i}\cdot z_{\text{mal},i}}{\|z_{\text{pos},i}{\|}_{2}\;\|z_{\text{mal},i}{\|}_{2}}|$$

where *B* is the batch size. Because both heads terminate in ReLU, the pooled vectors are non-negative and the cosine lies in [0,1]; minimizing it forces the two heads to activate on complementary channels. The weighting coefficient *λ*_orth_ controls the decoupling strength; after evaluating *λ*_orth_ ∈ {0.0,0.1,0.2,0.3,0.4,0.5}, we select *λ*_orth_ = 0.3 for the best balance between detection accuracy and feature decoupling (Section 4.4).

**Figure 3:**
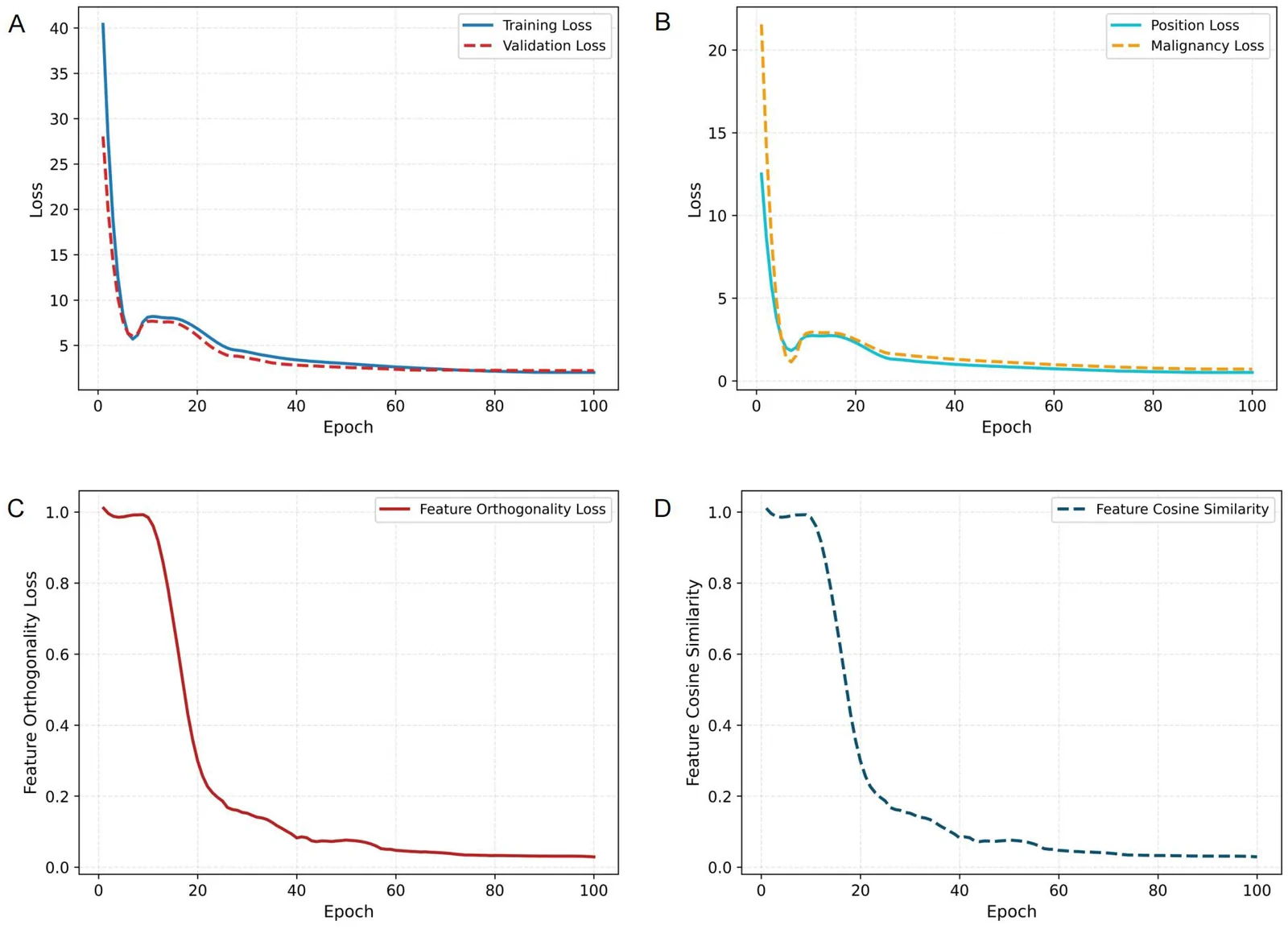
Loss convergence at *λ*_orth_=0.3. **(A)** Total loss, **(B)** Position vs. Malignancy loss, **(C)** $L_{\text{orth}}$, **(D)** Cosine similarity. Curves are smoothed via Savitzky–Golay filtering.

Orthogonality (zero correlation) is a necessary but not sufficient condition for statistical independence: independence always implies zero correlation, whereas the converse holds only under specific distributional assumptions such as joint Gaussianity, for which $\;I=-\frac{1}{2}\text{log }(1-\rho^{2})$ (48). Therefore, the proposed loss should be understood as a lightweight, second-order surrogate for mutual-information minimization; it removes linear dependence but cannot eliminate higher-order nonlinear dependence. We deliberately chose this formulation over heavier alternatives [e.g., MINE (37), CLUB (40)] because it adds no trainable parameters, produces stable gradients, and optimizes jointly with the detection losses without observable conflict (**Figure 3**).

**Figure 4:**
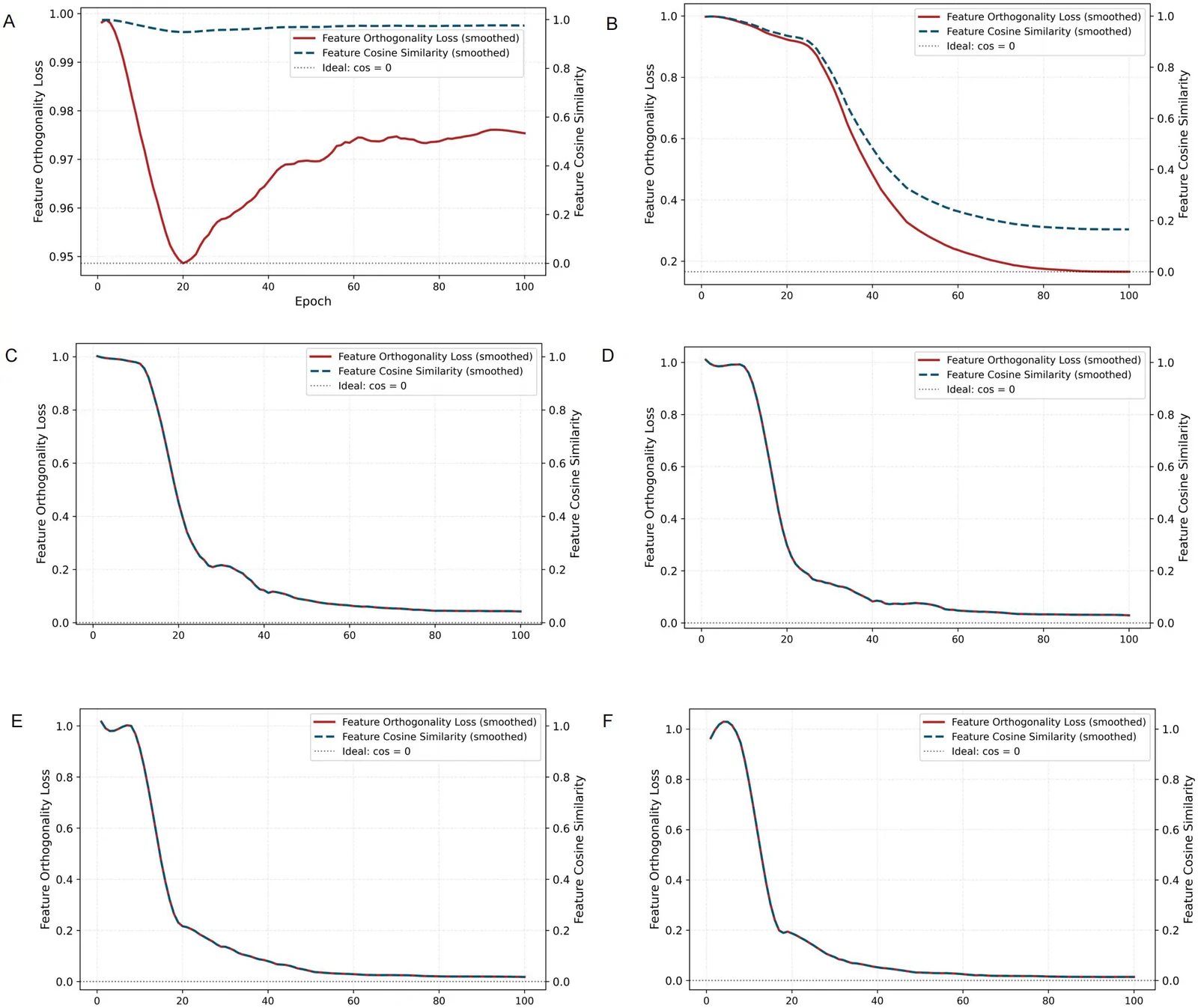
Training trends of $L_{\text{orth}}$ and cosine similarity. **(A–F)**: *λ*_orth_ = 0.0–0.5.

Empirically, the surrogate is effective: over training, the mean channel-wise Pearson correlation between *f*_pos_ and *f*_mal_ drops from 0.806 to 0.055 (**Table 2**) and the inter-stream cosine similarity converges to near zero (**Figure 4**), indicating that the linear redundancy between the two heads is substantially reduced. Capturing residual nonlinear dependence with kernel-based or variational criteria is left as future work.

**Table 2:** Feature decoupling metrics between *f*_pos_ and *f*_mal_.

a. Channel-wise Pearson correlation ( $\lambda_{\text{orth}}=0.3$)		
Metric	Epoch 5	Epoch 100
Mean r	0.8059	0.0552
Std	0.2623	0.7097
Mean |*r*|	0.8356	0.6368
r>0.3 (strongly +)	96.4%	40.0%
|r|≤0.3 (near-zero)	0.6%	18.4%
r<−0.3 (strongly –)	3.0%	41.6%
b. Nonlinear dependence measures		
Measure	$\lambda_{\text{orth}}=0.0$	$\lambda_{\text{orth}}=0.3$
Distance correlation	0.979	0.895
Linear CKA	0.959	0.830
Kernel (RBF) CKA	0.912	0.692

Panel (a): channel-wise Pearson correlation statistics at training Epoch 5 vs. Epoch 100 (*λ*_orth_=0.3). Panel (b): nonlinear cross-stream dependence measures comparing *λ*_orth_=0.0 and *λ*_orth_=0.3 on the validation set (lower indicates weaker dependence). *r* denotes the Pearson correlation coefficient.

## 4 Experiments and results

### 4.1 Experimental setup

Detection performance is evaluated using multi-threshold mean Average Precision (mAP), defined as the arithmetic mean of AP values at Intersection over Union (IoU) thresholds {0.4,0.5,0.6,0.7}. Mean AP is computed from unrounded per-class values, and all numerical values shown in relevant tables are rounded. Per-category F_1_, Recall, and Precision at IoU=0.5 are also reported. Differences between two AP or mAP values are absolute and are quoted in percentage points (pp); percentages are reserved for proportions such as recall, precision, and class or size shares. The evaluation metrics are formalized in Equations 20–23.

(20) $$\text{mAP}=\frac{1}{4}(\text{AP}@0.4+\text{AP}@0.5+\text{AP}@0.6+\text{AP}@0.7)$$

(21) Precision=TP/(TP+FP)

(22) Recall=TP/(TP+FN)

(23) $${\text{F}}_{1}=2\cdot \frac{\text{Precision}\cdot \text{Recall}}{\text{Precision}+\text{Recall}}$$

**Table 3** summarizes the complete training configuration and the inference-time complexity of both the baseline CenterNet and the proposed MADTHD. All experiments use PyTorch 1.8 with CUDA 11.1 on a single NVIDIA RTX 3060 (12GB). A fixed global seed (11) with deterministic cuDNN ensures reproducibility; statistical reliability is assessed via paired bootstrap (Section 4.5). For every model and ablation variant, we report the checkpoint with the lowest validation loss. No hard early-stopping rule was imposed: each run completed the full 100-epoch cosine-annealing schedule, and the best checkpoint was selected retrospectively. MADTHD adds only 0.12M parameters and 3.93GFLOPs over the baseline, and both models exceed 30FPS on a consumer-grade GPU.

**Table 3:** Training configuration and inference efficiency.

Item	Value
**(a) Training Configuration**			
Backbone / input / output	ResNet50 (ImageNet) / 512 × 512 / 128 × 128
Optimizer	Adam (β1 = 0.9, β2 = 0.999), weight decay = 0
Learning rate	5 × 10^−4^
Schedule	Freeze epochs 1–20, fine-tune 21–100
Augmentation	Random scale, h-flip, HSV jitter
Loss weights	α = 0.1, λ_orth_ = 0.3
Seed / reproducibility	11, deterministic cuDNN; paired bootstrap (2,000 ×)
Model selection	Best validation-loss checkpoint (full 100-epoch run)
**(b) Inference Efficiency (RTX 3060)**			
Model	CenterNet / MADTHD
Params (M)	32.66 / 32.78
GFLOPs	70.18 / 74.11
FPS / Latency	59.7/16.74 ms / 56.6/17.66 ms

### 4.2 Ablation study: contribution of each component

To quantify the incremental contribution of each MADTHD component, we started from CenterNet and added one component at a time, keeping the data split, schedule, and seed fixed (**Table 4**).

**Table 4:** Component-wise ablation on TN5000. Each row’s ∆ is computed relative to M0.

#	Configuration	Dual-head	Dilated	$L_{orth}$	mAP	∆(pp)
M0	CenterNet (baseline)	–	–	–	0.7579	—
M1	+ dual-head (only standard 3×3 convolution)	✓	–	–	0.7641	+0.62
M2	+ dilated convolutions(3×3 + dilation rate=2,4)	✓	✓	–	0.7540	−0.39
M3	+ L_orth_ (full MADTHD)	✓	✓	✓	0.7840	+2.61

Structural decoupling alone (M0 to M1) yields a modest gain (+0.62 pp). Adding dilated convolutions without the orthogonality constraint (M2) lowers mAP to 0.7540, 0.39 pp below the original baseline, whereas the same enlarged receptive field together with the orthogonality loss (M3) gives the full +2.61 pp improvement; the step from M2 to M3 (+3.00 pp) is the largest single increment in the table. The same contrast is visible across the other two ablations: with $L_{\text{orth}}$ active, the dilated Malignancy Head reaches 0.7840 against 0.7774 for the undilated one (**Table 5a**), whereas with $L_{\text{orth}}$ inactive dilation lowers mAP from 0.7641 to 0.7540 (rows M1 and M2 above). The enlarged receptive field is thus useful in combination with the decorrelation constraint.

**Table 5:** Receptive-field ablation.

Configuration	Cumul. RF	mAP	AP@0.5
(a) Malignancy-Head dilation rates			
{1,1} (no dilation)	5×5	0.7774	0.8144
{2,4} (proposed)	11×11	0.7840	0.8290
{4,8}	19×19	0.7868	0.8227
(b) Position-Head receptive field			
1×(3×3)	3×3	0.7530	0.8034
2×(3×3) (proposed)	5×5	0.7840	0.8290
3×(3×3)	7×7	0.7618	0.8015
2×(3×3*, r* = 2)	9×9	0.7696	0.8116

Panel (a): Malignancy-Head dilation rates with the Position Head fixed at 2×(3×3). Panel (b): Position-Head receptive field with the Malignancy Head fixed at {2,4}. Cumul. RF denotes the receptive field of each head’s shared trunk: the stacked 3×3 layers of the Position Head, and the base plus dilated branches of the Malignancy Head.

### 4.3 Ablation study: receptive-field configuration

To verify that the asymmetric receptive-field design is justified rather than arbitrary, we varied one architectural factor at a time with *λ*_orth_=0.3 and all other settings fixed (**Table 5**).

For the Malignancy Head (**Table 5a**), the no-dilation baseline {1,1} yields the lowest mAP (0.7774) and AP@0.5 (0.8144), so a standard receptive field is the weakest of the three settings. The two dilated settings are close: {4,8} attains the highest mAP of the panel (0.7868) and the proposed {2,4} follows at 0.7840. We retain {2,4} as a practical trade-off rather than as an optimum: the {2,4} configuration yields comparable inference speed with lower memory cost, and its 11×11 cumulative receptive field is closer to the scale of the nodules in TN5000 than the 19×19 field of {4,8}, with less exposure to the gridding artifacts associated with large dilation rates.

For the Position Head (**Table 5b**), the proposed two-layer 3×3 stack (5×5 RF) performs best among the four settings tested: a single layer under-contextualizes (−3.1 pp), while enlarging the RF via a third layer or dilation blunts the localization peak of small nodules. Taken together, the two panels show that the configuration best suited to the classification branch is not the one best suited to the localization branch, which is the observation the asymmetric design is built on.

### 4.4 Feature orthogonality loss and decoupling validation

To assess the effectiveness of $L_{\text{orth}}$ and select its weight, we evaluated *λ*_orth_ ∈ {0.0,0.1,0.2,0.3,0.4,0.5} with all other settings fixed. The case *λ*_orth_=0.0 retains the dual-head architecture but removes the decorrelation constraint.

**Figure 5:**
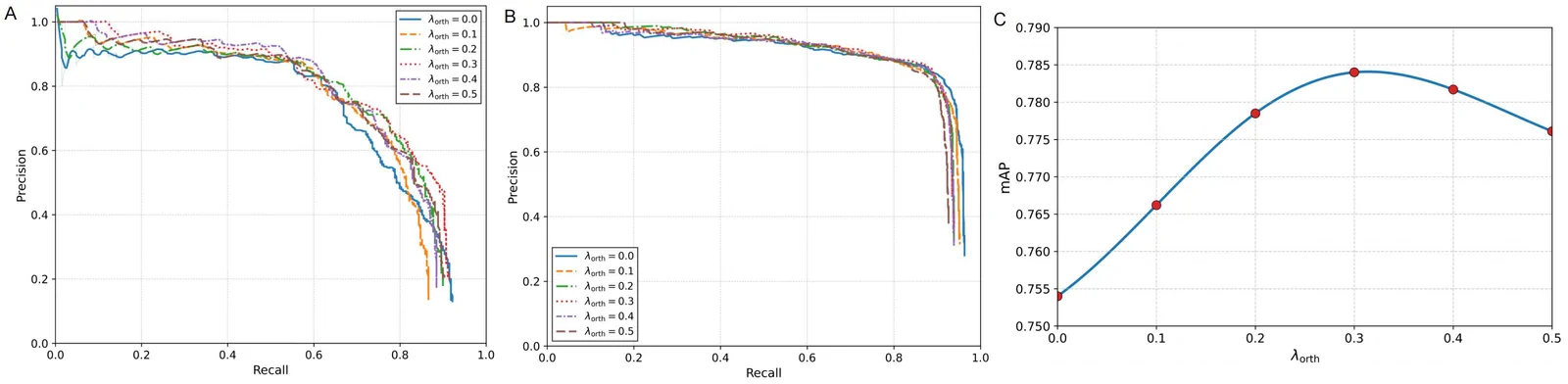
Effect of *λ*_orth_ on detection performance. **(A)** Precision–recall curves for benign nodules at IoU=0.5. **(B)** Precision–recall curves for malignant nodules. **(C)** Overall mAP as a function of *λ*_orth_.

As shown in **Table 6** and **Figure 5C**, mAP follows an inverted-U curve: it rises monotonically from 0.7540 (*λ*_orth_=0.0) to 0.7840 (*λ*_orth_=0.3), then declines at 0.4 and 0.5 due to gradient conflict between the orthogonality and detection objectives (31). Any positive *λ*_orth_ improves over *λ*_orth_=0.0, confirming the necessity of the constraint. The precision–recall curves at IoU=0.5 (**Figures 5A,B**) are consistent: Across both categories, *λ*_orth_=0.3 yields the best overall precision–recall curves. Across all configurations, benign AP remains below malignant AP, consistent with the class-imbalance analysis in Section 3.1.2.

**Table 6:** Detection performance under different values of *λ*_orth_.

*λ*_orth_	Category	AP				mAP	Note
		@0.4	@0.5	@0.6	@0.7		
0.0	BN	0.7332	0.7235	0.6728	0.4544	0.7540	No constraint
	MN	0.8991	0.8921	0.8662	0.7904		
	Mean AP	0.8162	0.8078	0.7695	0.6224		
0.1	BN	0.7488	0.7278	0.6918	0.4993	0.7662	Weak
	MN	0.8955	0.8875	0.8720	0.8069		
	Mean AP	0.8222	0.8077	0.7819	0.6531		
0.2	BN	0.7654	0.7505	0.7118	0.5586	0.7785	Moderate
	MN	0.8944	0.8817	0.8648	0.8009		
	Mean AP	0.8299	0.8161	0.7883	0.6797		
**0.3**	BN	0.7832	0.7737	0.7285	0.5247	0.7840	optimal
	MN	0.8958	0.8843	0.8654	0.8162		
	Mean AP	0.8395	0.8290	0.7969	0.6704		
0.4	BN	0.7722	0.7560	0.7211	0.5667	0.7817	Strong
	MN	0.8845	0.8771	0.8661	0.8098		
	Mean AP	0.8284	0.8165	0.7936	0.6882		
0.5	BN	0.7734	0.7540	0.7199	0.5623	0.7761	Over-strong
	MN	0.8793	0.8683	0.8572	0.7938		
	Mean AP	0.8263	0.8112	0.7886	0.6781		

#### 4.4.1 Training dynamics and convergence

**Figure 4** tracks $L_{\text{orth}}$ and the inter-stream cosine similarity across epochs. When *λ*_orth_=0.0, cosine similarity remains near 1 throughout, confirming the necessity of the constraint. At *λ*_orth_=0.1, both curves trend downward but do not converge, indicating a transitional state. For *λ*_orth_≥0.2, the two curves align closely and converge to near zero, indicating that the constraint converges stably and that the two pooled descriptors become close to orthogonal.

**Figure 3** shows all loss components at the selected *λ*_orth_=0.3. The Position and Malignancy Head losses converge independently, and the descent of $L_{\text{orth}}$ is synchronized with both, confirming the absence of severe gradient conflict. Training and validation losses track each other closely, indicating no pronounced overfitting.

#### 4.4.2 Quantitative and qualitative verification of feature decoupling

**Figure 6:**
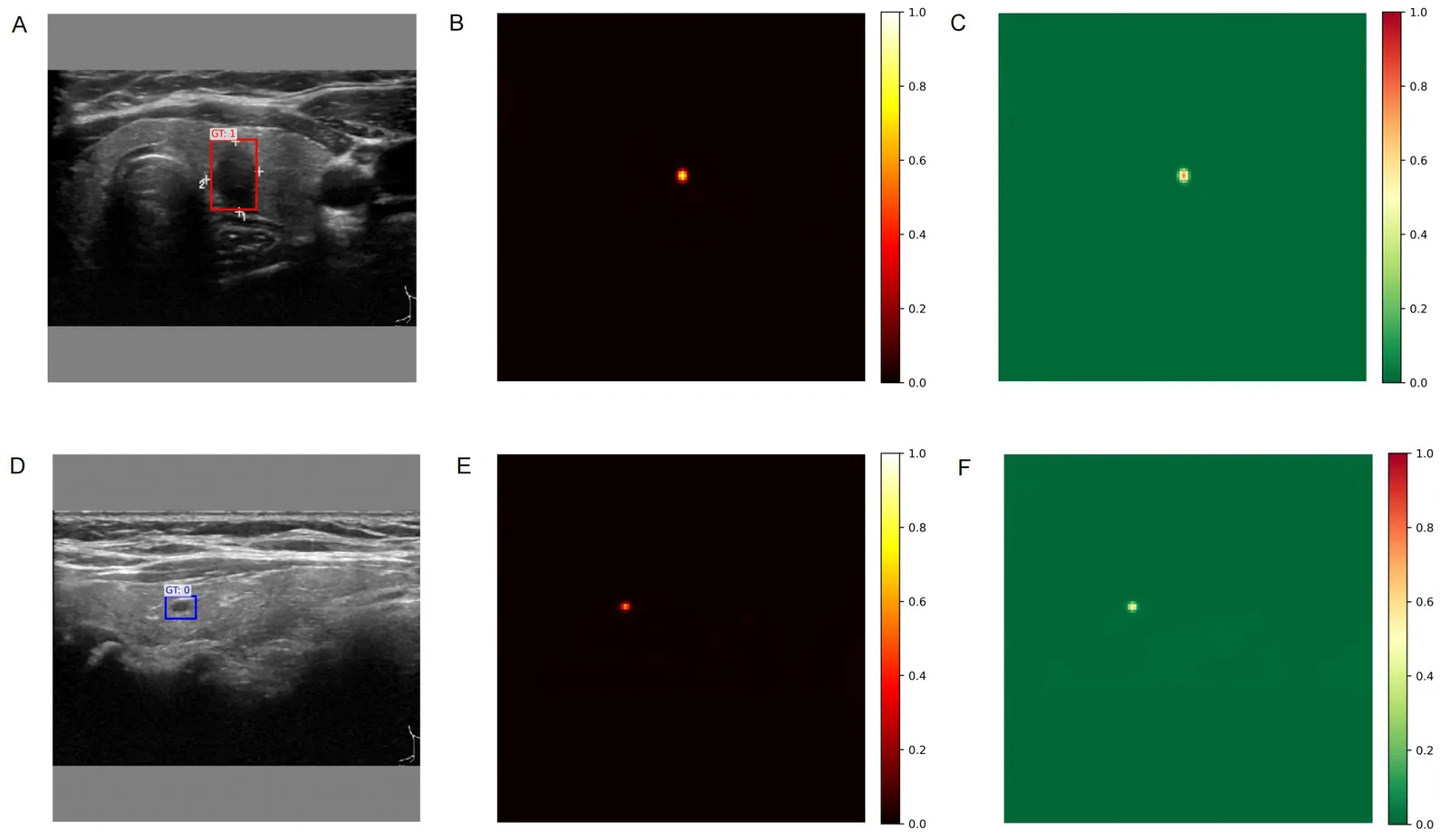
Dual-task heatmaps at *λ*_orth_=0.3. **(A–C)** Malignant: input, position heatmap, malignancy heatmap. **(D–F)** Benign: same layout.

Dual-heatmap visualization. **Figure 6** compares the dual heatmaps at *λ*_orth_=0.3. The Position Head produces focused, approximately circular responses centered on the nodule regardless of class, reflecting its category-agnostic design. The Malignancy Head exhibits more diffuse, class-dependent activation extending into peritumoral regions of malignant nodules, capturing features such as microcalcifications and spiculated margins. The two heatmaps thus encode clearly different spatial information for the same input.

**Figure 7:**
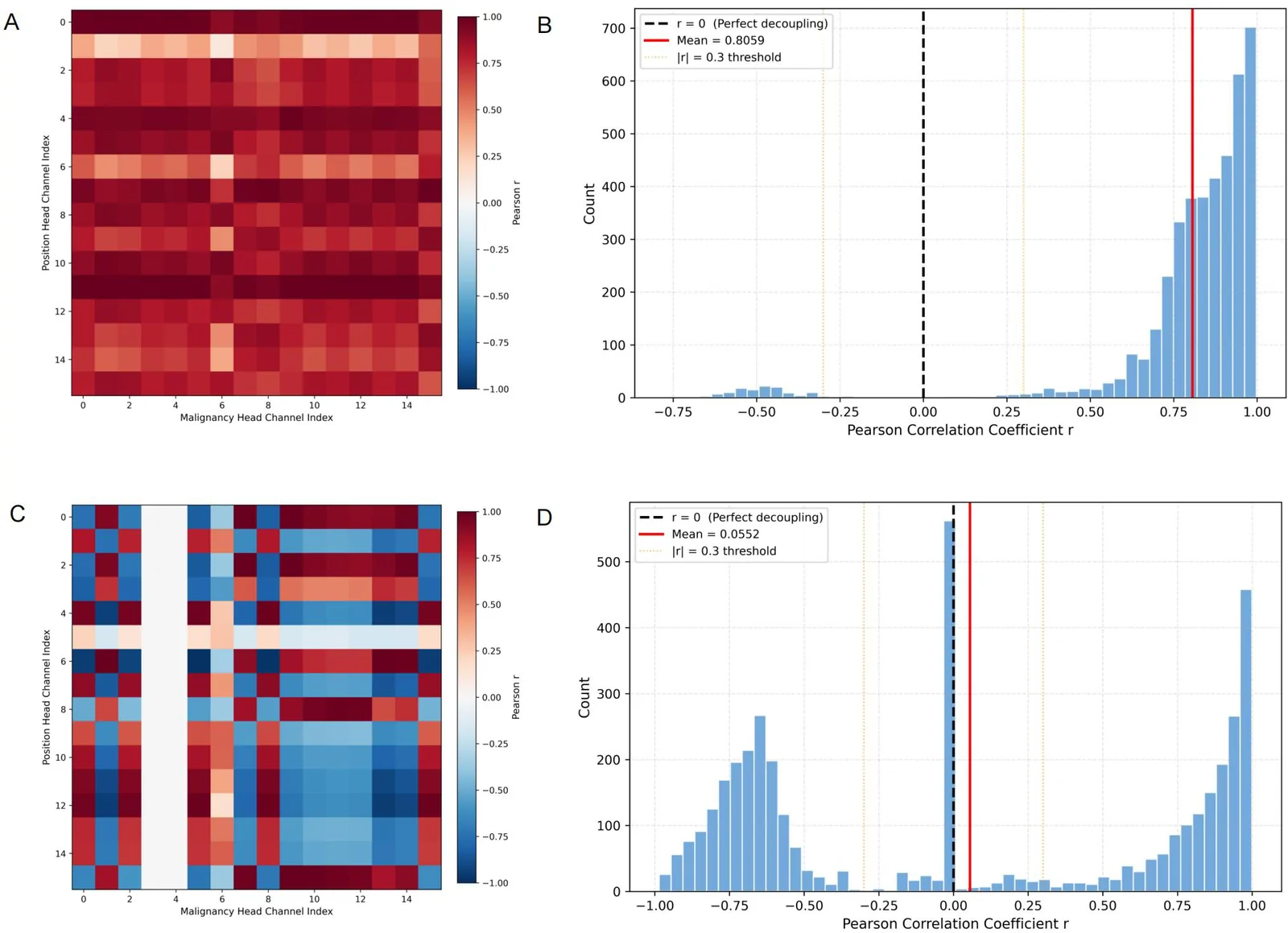
Feature correlation at Epoch 5 vs. Epoch 100. **(A, B)** Epoch 5: correlation heatmap and distribution histogram; **(C, D)** Epoch 100: correlation heatmap and distribution histogram.

Feature correlation and independence analysis. To quantify the decoupling effect, **Table 2** reports both channel-wise Pearson correlation statistics (computed over 50 validation samples) and nonlinear dependence measures between *f*_pos_ and *f*_mal_. **Figure 7** visualizes the correlation evolution.

Between Epoch 5 and Epoch 100 (**Table 2a**), the mean Pearson correlation drops from 0.806 to 0.055 and the mean absolute correlation from 0.836 to 0.637, the proportion of strongly positive pairs (*r>*0.3) falls from 96.4% to 40.0%, and near-zero pairs rise from 0.6% to 18.4%. The converged distribution (**Figures 7C,D**) contains positive, negative, and near-zero pairs, indicating diversified, complementary channel activations rather than a uniformly coupled representation. Because Pearson correlation captures only linear dependence, **Table 2b** additionally reports three nonlinear independence measures. All decrease once $L_{\text{orth}}$ is enabled, with kernel CKA showing the most notable drop from 0.912 to 0.692, confirming that the reduction in inter-stream dependence extends beyond linear correlation. Consistent with the second-order nature of the loss (Section 3.5.4), we interpret this as stronger decorrelation rather than exact statistical independence. The residual nonlinear dependence (e.g., distance correlation of 0.895) confirms that the second-order surrogate does not achieve full statistical independence, motivating future work with stronger independence criteria.

### 4.5 Detection performance and model comparison

**Table 7** compares MADTHD (*λ*_orth_=0.3) with the baseline CenterNet on the TN5000 test set. Overall mAP increases from 0.7579 to 0.7840 (+2.61 pp). The gain is most pronounced for malignant nodules: AP@0.5 rises from 0.8566 to 0.8843 (+2.77 pp), recall improves by 2.46 pp, and precision by 2.02 pp, consistent with the Malignancy Head’s multi-scale design. Benign AP@0.5 improves by 3.72 pp; the one exception is benign AP@0.7, which is marginally lower (0.5247 vs. 0.5468), reflecting greater boundary ambiguity under strict IoU.

**Table 7:** Per-category comparison: MADTHD vs. baseline CenterNet on TN5000.

Method	Category	AP				mAP	F_1_	Recall	Precision
		@0.4	@0.5	@0.6	@0.7				
MADTHD	BN	0.7832	0.7737	0.7285	0.5247	–	0.67	53.53%	87.80%
	MN	0.8958	0.8843	0.8654	0.8162	–	0.78	67.58%	92.51%
	Mean AP	0.8395	0.8290	0.7969	0.6704	0.7840	–	–	–
CenterNet	BN	0.7365	0.7365	0.6792	0.5468	–	0.66	52.79%	87.12%
	MN	0.8728	0.8566	0.8416	0.7930	–	0.76	65.12%	90.49%
	Mean AP	0.8046	0.7965	0.7604	0.6699	0.7579	–	–	–

To confirm that the improvement is not an artifact of test-set sampling, the uncertainty was quantified by a paired bootstrap analysis at the image level. The 1,000 test images were resampled with replacement 2,000 times (seed 11); within each resample, the same image list was used for both models, preserving the pairing, and the full mAP was recomputed. Confidence intervals are percentile bootstrap intervals, taken as the empirical 2.5th and 97.5th percentiles of the resampling distribution. As shown in **Table 8**, the percentile 95% interval for the paired difference ∆ is [0.0034, 0.0453]. This interval strictly excludes zero, MADTHD exceeds the baseline in 98.8% of the resamples, and the two-sided bootstrap *p*-value (derived directly from the resampling distribution) is 0.025.

**Table 8:** Paired bootstrap significance test. Δ= mAP(MADTHD)–mAP(CenterNet); two-sided p=0.025.

Model	mAP (95% CI)	Δ mAP (95% CI)
CenterNet	0.7579 [0.7264, 0.7932]	0.0261 [0.0034, 0.0453]
MADTHD	0.7840 [0.7543, 0.8147]	

#### 4.5.1 Comparison with mainstream detectors

**Table 9** compares MADTHD with seven mainstream detectors, all trained on the same TN5000 split for 100 epochs and scored with a single shared evaluation script. Each model’s architecture, input resolution, and optimization recipe are listed alongside its detection results for full reproducibility.

**Table 9:** Comprehensive comparison on TN5000.

Method	Year	Backbone/Neck	Optim./LR	Input	AP@0.4	AP@0.5	AP@0.6	AP@0.7	mAP
Faster R-CNN (7)	2015	ResNet50/–	Adam/10^−4^	600	0.7760	0.7720	0.7256	0.5625	0.7090
YOLOv5-s (49)	2020	CSPDarknet/PAFPN	SGD/10^−2^	640	0.7557	0.7460	0.7124	0.5685	0.6957
RetinaNet (50)	2017	ResNet50/FPN	Adam/10^−4^	600	0.8140	0.8022	0.7770	0.6681	0.7653
CenterNet (9)	2019	ResNet50/–	Adam/5×10^−4^	512	0.8046	0.7965	0.7604	0.6699	0.7579
DETR (28)	2020	ResNet50/Transformer	AdamW/10^−4^	800	0.8520	0.8162	0.6839	0.4327	0.6962
MADTHD (ours)	—	ResNet50/–	Adam/5×10^−4^	512	0.8395	0.8290	0.7969	0.6704	0.7840
YOLOX-s (12)	2021	CSPDarknet/PAFPN	SGD/10^−2^	640	0.8618	0.8517	0.8202	0.7172	0.8127
FCOS (13)	2019	ResNet50/FPN	Adam/3×10^−4^	640	0.8633	0.8554	0.8311	0.7264	0.8191

Among methods sharing the same backbone family (ResNet50, single-scale, no FPN), MADTHD achieves the highest mAP (0.7840), surpassing CenterNet (+2.61 pp) and Faster R-CNN by 7.50 pp. Despite employing feature pyramid necks (FPN or PAFPN), RetinaNet (0.7653) and YOLOv5-s (0.6957) both remain below MADTHD. YOLOX-s (0.8127) and FCOS (0.8191) achieve higher absolute mAP, which is expected: their gains derive from multi-scale feature pyramids (PAFPN/FPN), stronger augmentation (mosaic, mixup), and larger input resolution, all of which are architectural advantages orthogonal to the head-level design studied here. The proposed modules are backbone- and neck-agnostic; integrating them into FPN-based detectors is a promising direction for future work. DETR achieves competitive AP at loose IoU (AP@0.4 = 0.8520) but collapses at strict thresholds (AP@0.7 = 0.4327, mAP=0.6962), consistent with its known slow convergence under limited epochs and weak small-object localization without FPN; we report it as a same-budget reference.

Although the overall mAP gain is moderate (+2.61 pp), it is statistically significant, obtained without additional training data and with negligible inference overhead, and more pronounced on the clinically relevant malignant category (AP@0.5 + 2.77 pp, recall +2.46 pp). Beyond the numerical gain, the principal contribution is the interpretable dual-task framework with task-separated heatmaps.

### 4.6 Analysis of visualized detection results

**Figure 8:**
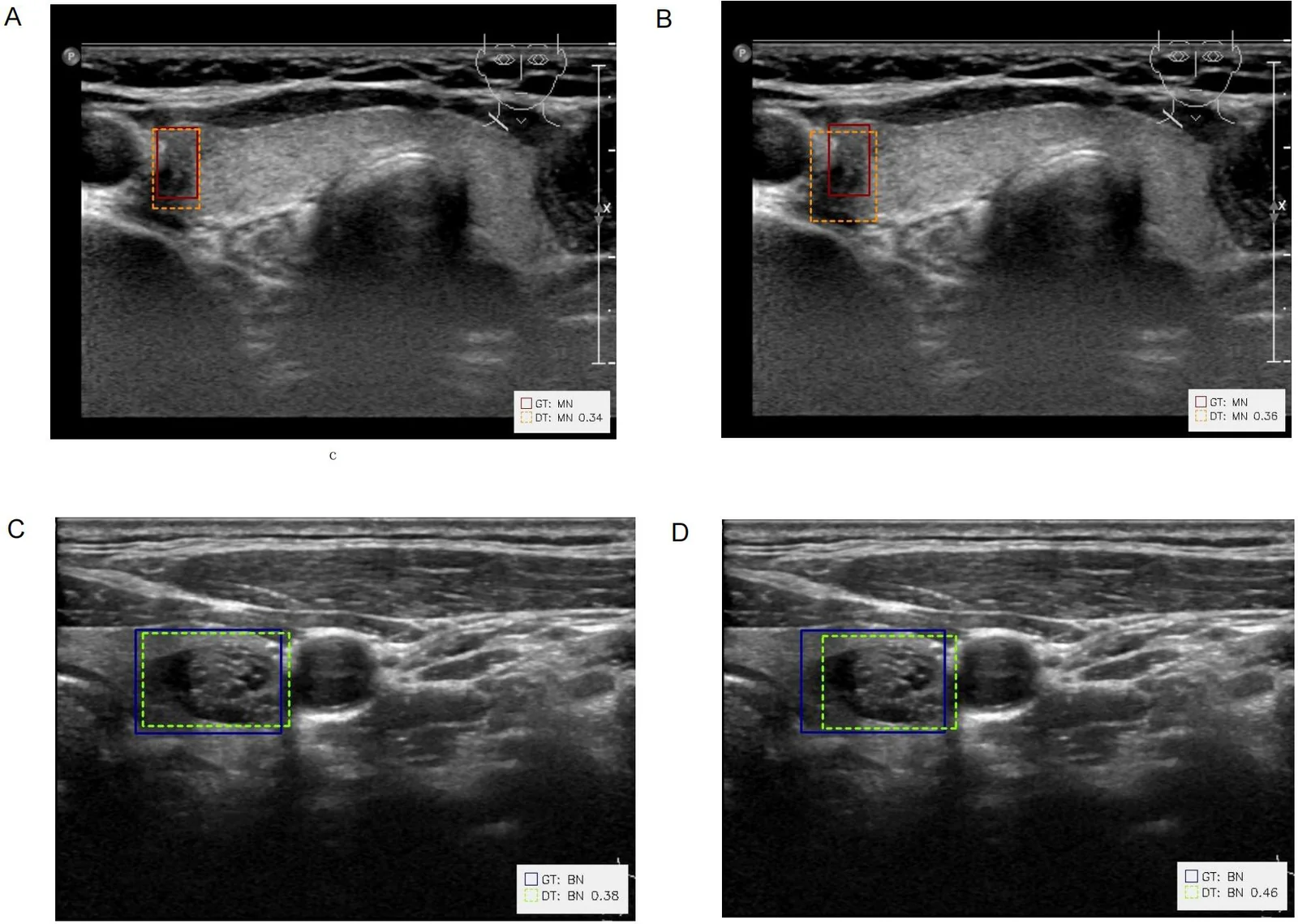
True positive detections. **(A, B)** Malignant: MADTHD vs. CenterNet. **(C, D)** Benign.

**Figure 9:**
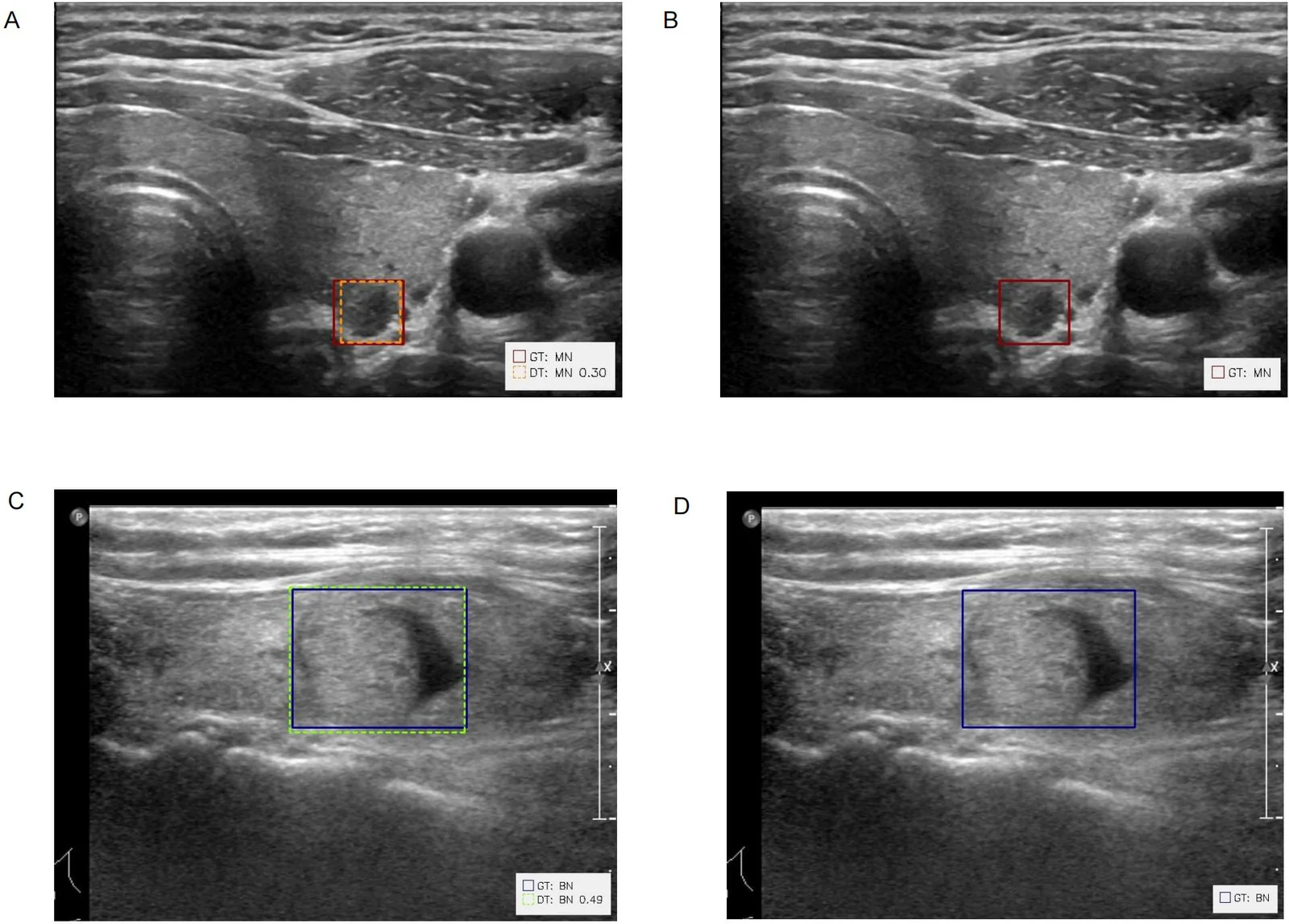
Recovery of baseline missed detections. **(A, B)** Malignant. **(C, D)** Benign.

**Figure 10:**
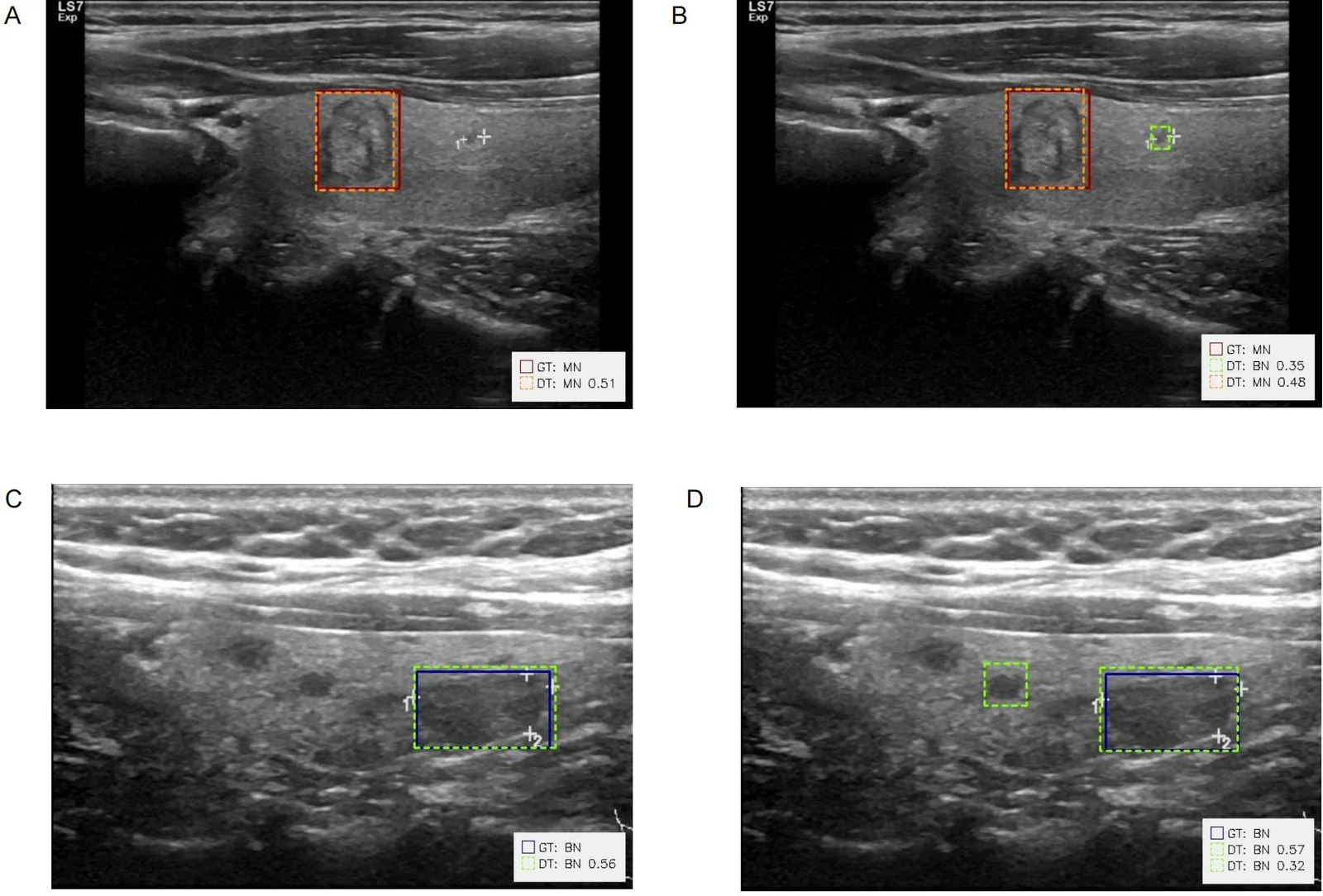
Suppression of baseline false positives. **(A, B)** and **(C, D)**: two cases.

**Figure 11:**
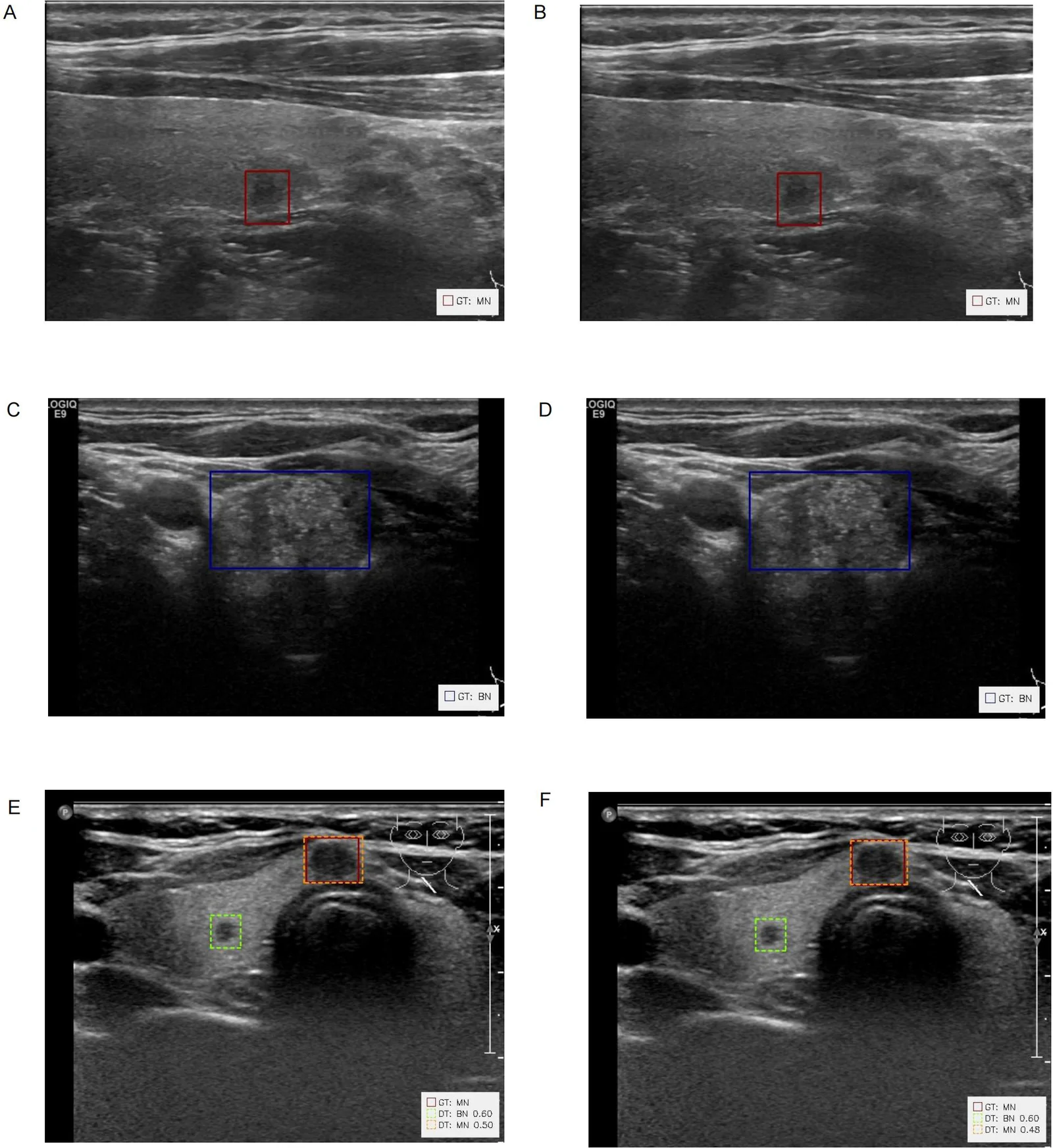
Failure cases. **(A, B)** Diminutive nodule. **(C, D)** Low contrast. **(E, F)** Mimicking anatomy.

To validate practical detection performance, we compare MADTHD with CenterNet on representative test images. Each predicted box is labeled “DT: [class] [score]” (confidence ≥0.3), where [class] indicates the predicted category (BN for benign, MN for malignant) and [score] is the predicted probability; benign/malignant DT boxes are green/orange dashed lines; Ground truth (GT) boxes are labeled “GT: [class]”, benign/malignant GT boxes are blue/red solid lines. Samples are selected to cover true positives, recovered misses, corrected false positives, and failure cases. Across **Figures 8**-**11**, the first column shows MADTHD outputs, and the second those of the CenterNet baseline.

#### 4.6.1 True positives and recovered detections

For malignant nodules (**Figures 8A, B**), MADTHD achieves IoU=0.7405 versus 0.4185 for the baseline. For benign nodules (C, D), MADTHD reaches IoU=0.8129 versus 0.4578, producing tighter boxes on well-defined margins.

In **Figure 9**, CenterNet’s confidence falls below threshold (0.2472 and 0.2998), while MADTHD achieves successful detections with IoU 0.8203 and 0.9272, recovering nodules that the baseline misses at the same threshold.

#### 4.6.2 Corrected false positives

Glandular shadow regions trigger false alarms in CenterNet (confidence 0.3491 and 0.3239), while MADTHD’s responses remain below threshold (0.2988 and 0.2674). The Malignancy Head’s larger receptive field enables better discrimination of true nodules from mimicking backgrounds.

#### 4.6.3 Failure analysis

Both models fail in three scenarios (**Figure 11**): (i) ultra-small nodules with sparse pixel-level information; (ii) low-contrast lesions whose gray-level distribution overlaps with surrounding tissue; (iii) high-mimicry anatomical structures that resemble nodules. These cases suggest that single-frame ultrasound may be insufficient for extreme corner cases; multi-frame fusion and clinical-prior-guided attention are promising future directions.

#### 4.6.4 Representative detections

**Figure 12:**
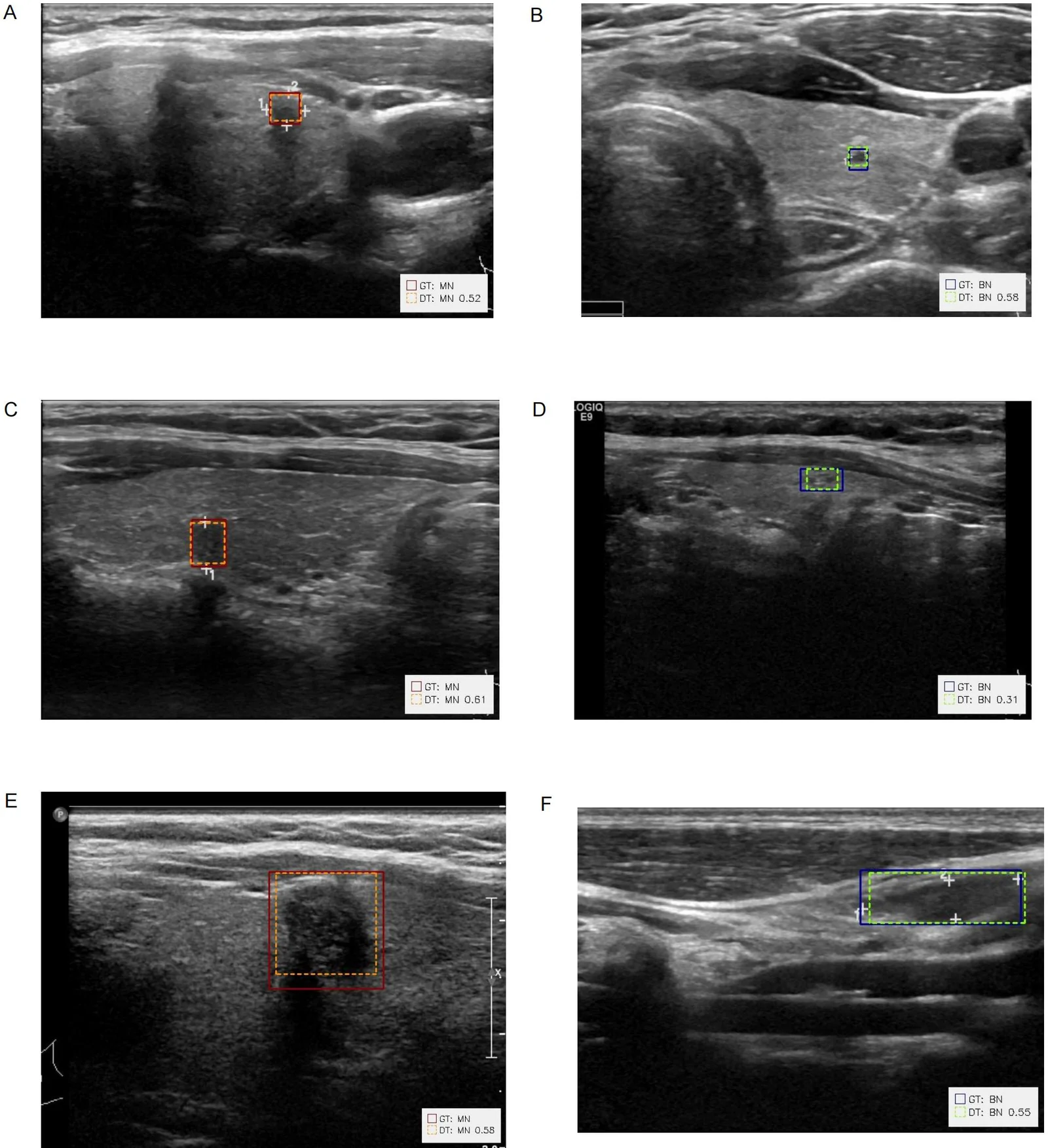
Representative detections by MADTHD across diverse clinical scenarios. **(A, C, E)** Malignant. **(B, D, F)** Benign.

MADTHD maintains reliable detection across diverse nodule morphologies, echo patterns, and imaging conditions in the majority of clinical scenarios, as shown in **Figure 12**, despite remaining challenges with ultra-low-contrast images.

### 4.7 External validation on TN3K

To assess generalization, we evaluated on the independent TN3K dataset (51) under a strict zero-shot transfer protocol: TN5000-trained models are tested on TN3K without fine-tuning. TN3K was originally released for nodule segmentation; the benign/malignant classification labels were subsequently provided by the dataset authors as part of the official public distribution (52) and annotated by experienced radiologists. TN3K was collected at a different institution from TN5000, which is what makes it informative as an external set.

**Figure 13:**
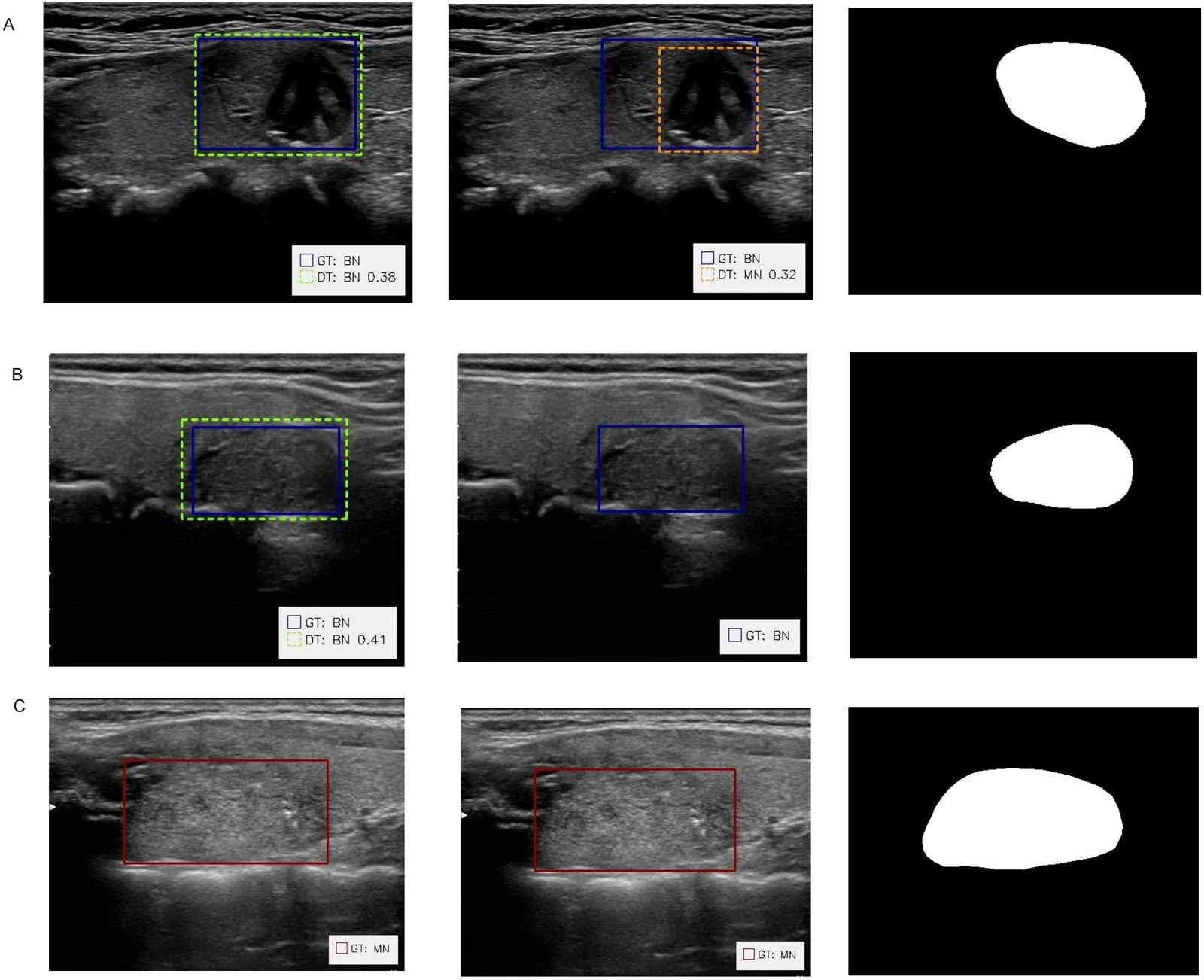
Representative zero-shot detection results on TN3K. Each row shows: the MADTHD prediction, the CenterNet prediction, and the TN3K segmentation mask. **(A)** MADTHD correctly localizes and classifies the nodule, while the baseline makes a classification error. **(B)** MADTHD recovers a nodule missed by the baseline. **(C)** Both models fail on a very low-contrast nodule under severe domain shift. DT/GT box colors follow **Figure 8**.

Evaluated sample and conversion procedure. The official TN3K test split of 614 images was used in full, and no image was excluded. Since TN3K provides pixel-level segmentation masks rather than bounding boxes, each mask was resized to the image resolution by nearest-neighbor interpolation where the two differed, binarized at an intensity of 127, and decomposed into connected components under 8-connectivity; the tight axis-aligned box of every component covering at least 30 pixels was retained. This yields 690 ground-truth boxes, of which 431 (62.5%) are benign and 259 (37.5%) malignant, distributed over 378 benign and 236 malignant images. Seventy images (11.4%) contain more than one box, and since the released labels are defined per image, every box in these images inherits the image-level label. The same image list and ground-truth files were used for both models, and the original masks are shown alongside the detection results in **Figure 13**.

Characterizing the shift. The two datasets differ in more than acquisition site and device. The benign fraction rises from roughly 29% in TN5000, where the benign-to-malignant ratio is about 1:2.5, to 62.5% here, so the class prior is close to inverted. The nodules are also larger: small objects, defined as in Section 3.1.2 by a relative area below 10%, account for 404 of the 690 TN3K boxes (58.6%) against 83.0% in TN5000, with 255 medium (37.0%) and 31 large (4.5%). Both properties work against a model trained on TN5000 and are reflected in the absolute scores below.

As shown in **Table 10**, both models experience an mAP drop to approximately 0.39, attributable to the shift characterized above: different institutions, devices and annotation protocols, an inverted class prior, a coarser nodule-size distribution, and boxes derived from segmentation masks rather than drawn as detection boxes. Despite this, MADTHD retains its advantage (+1.04 pp), and the improvement grows with stricter IoU: ∆AP@0.7=+2.21 pp. On this smaller set, the paired bootstrap yields a ∆ mAP of 0.0104 (95% CI: [−0.0144, 0.0341]). The interval includes zero, so the difference is not resolved statistically on this smaller set; the point estimate and the per-threshold pattern nonetheless favor MADTHD.

**Table 10:** Zero-shot external validation on TN3K with per-class AP.

Method	Category	AP@0.4	AP@0.5	AP@0.6	AP@0.7	mAP
CenterNet	BN	0.4563	0.3827	0.3027	0.2062	–
	MN	0.5248	0.4840	0.4212	0.3077	–
	Mean AP	0.4905	0.4334	0.3619	0.2570	0.3857
MADTHD	BN	0.4427	0.3808	0.3120	0.2360	–
	MN	0.5333	0.4943	0.4477	0.3221	–
	Mean AP	0.4880	0.4375	0.3798	0.2791	0.3961
∆ (Mean, pp)		−0.25	+0.41	+1.79	+2.21	+1.04

## 5 Discussion

The experiments on TN5000 demonstrate that MADTHD consistently outperforms the baseline CenterNet across most operating points, with an overall mAP improvement of 2.61 pp that is statistically significant (two-sided paired bootstrap *p* = 0.025). The core advantages operate at two levels. First, decoupling the localization objective from classification sharpens the response to small nodules, which is reflected in higher recall. Second, the Malignancy Head’s dilated convolutions capture peritumoral texture and boundary cues, suppressing false positives from mimicking anatomical structures and improving precision. The training dynamics (**Figures 3**, **4**) confirm that the feature orthogonality loss converges without gradient conflict, and the channel-wise correlation analysis (**Table 2**) shows that linear inter-head redundancy is substantially reduced.

### 5.1 Analysis of challenging scenarios

The benign class remains the principal challenge: despite MADTHD improving benign AP@0.5 from 0.7365 to 0.7737, the benign-to-malignant imbalance (≈1:2.5) provides a weaker learning signal, and many benign nodules exhibit iso- to hypo-echoic patterns whose margins blend into surrounding tissue, corresponding to the low-contrast and high-mimicry failure modes in **Figure 11**. Class-balanced training strategies offer a natural avenue for further improvement.

As analyzed in the comparison with mainstream detectors (**Table 9**), the residual gap to FPN-based methods such as FCOS and YOLOX stems from architectural components (multi-scale feature pyramids, stronger augmentation, larger input resolution) that are orthogonal to the head-level contribution studied here; the proposed dual-task decoupling and feature-orthogonality modules are backbone- and neck-agnostic and could in principle be combined with such detectors in future work.

### 5.2 Comparison with previous studies

Early deep-learning studies largely treated detection, segmentation, and classification of thyroid nodules as separate problems. A recurring observation is that localization and characterization place different demands on the feature representation, yet most joint detection-and-classification pipelines still rely on a single shared head. MADTHD departs from this convention by making the heterogeneity explicit at the head level with a feature-orthogonality constraint, which represents a design axis complementary to segment-then-classify cascades and multi-task loss-weighting schemes.

Because prior studies use different datasets, annotation protocols, and metrics, we compare methodologically rather than by absolute scores. Within our controlled benchmark, the improvement is obtained without additional data, a deeper backbone, or measurable inference overhead (**Table 3**), so the gain follows from the head-level changes rather than from added capacity or data. Moreover, in contrast to the opaque regression branches of conventional decoupled heads, MADTHD yields two human-readable heatmaps that mirror the clinical localize-then-characterize workflow, offering interpretability that purely accuracy-driven models do not provide. Together with the consistent ordering on TN3K under zero-shot transfer (Section 4.7), these findings suggest that explicit task decoupling is a transferable, low-cost design principle for medical lesion detection.

### 5.3 Limitations and future work

Several factors delimit the scope of this study and define directions for future work.

Data dependency: The primary experiments were conducted on TN5000, a single publicly available dataset from one institution; although zero-shot external validation on TN3K (Section 4.7) shows the same relative ordering under distribution shift, the absolute cross-domain performance degrades substantially, so validation across additional centers, scanners, and acquisition protocols is needed before clinical deployment.

Annotation: Each TN5000 image is annotated with a single nodule, so multifocal cases and inter-observer variability are not explicitly modeled, and the TN3K evaluation converts segmentation masks to bounding boxes, introducing annotation-protocol discrepancies that contribute to the measured gap; boxes derived from masks are also tighter than manually drawn detection boxes, which penalizes both models at strict IoU thresholds.

Class imbalance: The benign-to-malignant ratio (≈1:2.5 in TN5000) provides a weaker learning signal for the benign class and may not reflect screening-population prevalence, leaving room for class-balanced training strategies.

Domain shift: Variation across devices, operators, and imaging settings is only partially probed and not fully resolved by the proposed method.

Accordingly, the present results should be read as evidence of algorithmic improvement on recognized public benchmarks rather than as validated clinical performance: translating these gains into clinical benefit, such as assisting radiologists or reducing unnecessary biopsies, will require prospective, multi-reader validation, which we intend to pursue together with extensions to stronger independence criteria, multi-nodule annotation, and dynamic multi-frame and multi-modal inputs (e.g., color Doppler and elastography).

## 6 Conclusions

This paper proposes MADTHD, a Malignancy-Aware Dual-Task Heatmap Decoupling method for joint detection and benign-malignant classification of thyroid ultrasound nodules. By separating class-agnostic localization (Position Head) from malignancy discrimination (Malignancy Head) and introducing a feature orthogonality loss, MADTHD separates the two clinical questions (“where is the nodule?” and “is it malignant?”), mirroring the sequential reasoning of radiologists. On TN5000, MADTHD improves mAP by 2.61 percentage points over CenterNet (malignant AP@0.5 reaching 0.8843), a gain that is statistically significant (two-sided paired bootstrap *p* = 0.025) and obtained without additional data or a deeper backbone. Ablation studies demonstrate that the asymmetric dual-head architecture and the feature orthogonality loss are mutually synergistic, effectively combining precise spatial localization with robust multi-scale malignancy discrimination. Furthermore, zero-shot evaluation on TN3K preserves the same relative ordering. Beyond the numerical gain, the dual-head design contributes interpretable, task-separated heatmaps, which we regard as the principal contribution of this work. Future work will extend validation to multi-center datasets, explore stronger independence criteria, and generalize the framework to three-dimensional ultrasound analysis.

## Data Availability

The original contributions presented in the study are included in the article/supplementary material. Further inquiries can be directed to the corresponding author.
